# The Mechanism of Transcription Factor Swi6 in Regulating Growth and Pathogenicity of *Ceratocystis fimbriata*: Insights from Non-Targeted Metabolomics

**DOI:** 10.3390/microorganisms11112666

**Published:** 2023-10-30

**Authors:** Hao Cong, Changgen Li, Yiming Wang, Yongjing Zhang, Daifu Ma, Lianwei Li, Jihong Jiang

**Affiliations:** 1 The Key Laboratory of Biotechnology for Medicinal and Edible Plant Resources of Jiangsu Province, School of Life Sciences, Jiangsu Normal University, Xuzhou 221116, China; conghao@jsnu.edu.cn (H.C.); 1020200018@jsnu.edu.cn (C.L.); 1020210013@jsnu.edu.cn (Y.W.); 1020210011@jsnu.edu.cn (Y.Z.); 2 Chinese Academy of Agricultural Sciences Sweet Potato Research Institute, Xuzhou 221131, China; daifuma@163.com

**Keywords:** *Ceratocystis fimbriata*, metabolomics, Swi6, virulence, sweet potato black rot

## Abstract

*Ceratocystis fimbriata* (*C. fimbriata*) is a notorious pathogenic fungus that causes sweet potato black rot disease. The APSES transcription factor Swi6 in fungi is located downstream of the cell wall integrity (CWI)-mitogen-activated protein kinase (MAPK) signaling pathway and has been identified to be involved in cell wall integrity and virulence in several filamentous pathogenic fungi. However, the specific mechanisms by which Swi6 regulates the growth and pathogenicity of plant pathogenic fungi remain elusive. In this study, the *SWI6* deletion mutants and complemented strains of *C. fimbriata* were generated. Deletion of Swi6 in *C. fimbriata* resulted in aberrant growth patterns. Pathogenicity assays on sweet potato storage roots revealed a significant decrease in virulence in the mutant. Non-targeted metabolomic analysis using LC-MS identified a total of 692 potential differentially accumulated metabolites (PDAMs) in the ∆*Cfswi6* mutant compared to the wild type, and the results of KEGG enrichment analysis demonstrated significant enrichment of PDAMs within various metabolic pathways, including amino acid metabolism, lipid metabolism, nucleotide metabolism, GPI-anchored protein synthesis, and ABC transporter metabolism. These metabolic pathways were believed to play a crucial role in mediating the growth and pathogenicity of *C. fimbriata* through the regulation of CWI. Firstly, the deletion of the *SWI6* gene led to abnormal amino acid and lipid metabolism, potentially exacerbating energy storage imbalance. Secondly, significant enrichment of metabolites related to GPI-anchored protein biosynthesis implied compromised cell wall integrity. Lastly, disruption of ABC transport protein metabolism may hinder intracellular transmembrane transport. Importantly, this study represents the first investigation into the potential regulatory mechanisms of *SWI6* in plant filamentous pathogenic fungi from a metabolic perspective. The findings provide novel insights into the role of *SWI6* in the growth and virulence of *C. fimbriata*, highlighting its potential as a target for controlling this pathogen.

## 1. Introduction

As one of the most important tuber crops in the world, sweet potato (*Ipomoea batatas* (L.) Lam.) is extensively cultivated, particularly in Asia and Africa, due to its high yield and rich content of starch and other beneficial nutrients for human consumption [1,2,3]. *C. fimbriata* is the causative agent of sweet potato black rot, a highly destructive storage disease that poses a significant threat to sweet potato crops, and this plant fungal pathogen exhibits a hemi biotrophic infection style, primarily targeting the nutrient tissues of sweet potatoes [4,5,6,7]. *C. fimbriata* not only infects sweet potato storage roots through wounds but also invades the vascular xylem, ultimately causing plant wilting and death [5,8]. *C. fimbriata* infection in sweet potato storage roots can induce the production of furanoterpenoid toxins, namely ipomeamarone, dehydroipomeamarone, and ipomeamaronol [3,9]. Consumption of sweet potatoes contaminated with these toxins can result in severe liver and lung damage in both humans and animals [4,10]. Currently, research on sweet potato black rot primarily focuses on the development of chemical reagents to combat *C. fimbriata*, the breeding of resistant sweet potato varieties, and comprehensive prevention and control measures in agricultural cultivation [5,11,12,13,14]. However, there is little research on the pathogenic mechanism of *C. fimbriata* to help develop effective prevention and control measures for sweet potato black rot.

Studying key pathogenic genes is crucial for understanding the pathogenesis of pathogenic fungi. It enables the identification of diagnostic markers and therapeutic targets, thereby providing a theoretical foundation for the prevention and treatment of fungal infections [15,16,17,18,19,20]. The APSES (Asm1p, Phd1p, Sok2p, Efg1p, and Stuap) protein family, characterized by a basic helix–loop–helix (bHLH) domain, consists of fungus-specific transcription factors that play crucial roles in various cellular processes [21,22]. In *Aspergillus fumigatus*, the APSES transcription factor MbsA regulates growth, spore wall architecture, Gliotoxin production, and virulence via the SakA signaling pathway [22]. The APSES protein Swi6 interacts with Mbp1 to form the MluI cell cycle box-binding complex, regulating mycelial differentiation and virulence in *Beauveria bassiana* [23,24]. The transcription factor CsXbp1 plays a role in appressoria formation and sclerotial development in *Ciboria shiraiana*. Additionally, the pathogenicity of CsXbp1 RNAi strain to tobacco is significantly weakened [25]. In the nematode-trapping fungus *Arthrobotrys oligospora*, AoPkc, AoSlt2, and AoSwi6 are involved in regulating trap formation and pathogenicity. Additionally, the deletion of genes encoding these proteins significantly impacts the changes in secondary metabolites [26]. The transcriptional cofactor FgSwi6 is indispensable for cellulose utilization and deoxynivalenol production in *Fusarium graminearum*. Furthermore, FgSwi6 plays a role in phenotypic alterations induced by *F*. *graminearum* virus 1 infection. The deletion of the *FgSWI6* gene significantly diminishes *F*. *graminearum* virulence on wheat plants by approximately 80% [27,28]. The deletion of Mstu1 or Swi6 in APSES transcription factor resulted in notable alterations in mycelial growth and morphology in *Magnaporthe oryzae*. Moreover, these mutants exhibited impaired ability to penetrate rice leaves [29,30]. However, the functionality of Swi6 in *C. fimbriata* has not been reported to date.

In this study, we aimed to investigate the growth and pathogenicity mechanisms of *C. fimbriata* through analysis of the metabolic profile changes resulting from *SWI6* gene deletion. The deletion of the *SWI6* gene in *C. fimbriata* resulted in the loss of infectivity towards sweet potato storage roots. Metabolomic analysis of *C. fimbriata* and the Δ*Cfswi6* identified 692 PDAMs across 13 categories, including organic acids and derivatives, lipids and lipid-like molecules, and organoheterocyclic compounds. The disruption of *SWI6* primarily reprogrammed metabolic pathways such as amino acid metabolism, GPI-anchored protein biosynthesis, and ABC transporters, which may lead to impaired growth and reduced virulence in *C. fimbriata*. Our survey offers a novel perspective for investigating the growth and virulence mechanism of *C. fimbriata*, and establishes a theoretical basis for the prevention and treatment of sweet potato black rot.

## 2. Materials and Methods

### 2.1. Strains, Sweet Potato Samples, and Culture Conditions

The wild-type strain utilized in this study was *C. fimbriata* BMPZ13, which was described in our previous studies [5]. The sweet potato cultivar ‘Shang 19’ was provided by the Sweet Potato Research Institute of the Chinese Academy of Agricultural Sciences (Xuzhou, China). To evaluate the fungal growth characteristics of the strains under investigation, complete medium (CM) and potato dextrose agar (PDA) were employed. The *CfSWI6* gene was disrupted using homologous recombination according to previously reported methods [29,31,32], and the strategies for targeted disruption and complementation were illustrated in Supplementary Figure S1. The mutants (∆*Cfswi6-3 and* ∆*Cfswi6-5*) and complementary mutant Δ*Cfswi6/CfSWI6* were preserved using PDA medium.

### 2.2. Vegetative Growth and Pathogenicity Assays of C. fimbriata

The wild-type strain of *C. fimbriata* (BMPZ13), *CfSWI6* gene knockout mutants (Δ*Cfswi6-3* and Δ*Cfswi6-5*), and the complemented strains (Δ*Cfswi6/CfSWI6*) were cultured on CM and PDA medium for 5 days at 27 °C. Mycelial blocks measuring 2 mm × 2 mm from CM solid medium were excised and inoculated onto CM liquid medium at 27 °C with a shaking speed of 160 rpm. Following incubation, the colony diameter, hyphal balls, and biomass of *C. fimbriata* were evaluated. Conidia of the tested strains from CM solid medium were collected and suspended at a concentration of 10^6^ conidia/mL. The suspension was injected into drilled sweet potato storage roots, followed by incubation at 27 °C in the dark for 25 days. The storage root of sweet potato was observed, and measurements were taken for weight loss rate and lesion area. All inoculation assays were conducted in triplicate and repeated three times.

### 2.3. Ipomeamarone Detection of Sweet Potato

A 100 g sample of sweet potato was collected from the lesion and surrounding area. The sample was ground into powder using liquid nitrogen and extracted with methanol. The methanol extract was concentrated, dissolved in water, and subjected to repeated extraction using methylene chloride. The resulting extract was analyzed using a Trace-1300 ISQ (Thermo Fisher, Waltham, MA, USA) gas chromatography-mass spectrometry (GC-MS) system equipped with an HP-5 column (30 m × 0.25 mm internal diameter × 0.25 μm). Helium was used as the carrier gas at a flow rate of 1.2 mL/min. The temperature was initially set at 40 °C for 3 min, then increased to 280 °C at a rate of 10 °C/min, and held for 10 min. Each sample was assessed using three biological replicates.

### 2.4. Ultra-High-Performance Liquid Chromatography-Mass Spectrometry Tests for C. fimbriata

The control group, referred to wild-type BMPZ13 (FWT), and the treatment group, referred to treatment mutant ∆*Cfswi6* (FMT), were obtained by culturing the strains in CM liquid medium at 27 °C for 2 days with a shaking speed of 160 rpm. To separate the mycelium and filtrate, the fermentation broth underwent filtration. The filtrate was then freeze-dried until a constant weight was reached. Six biological replicates are required for each sample.

A 400 μL methanol/water (4:1, *v*/*v*) solution with 0.02 mg/mL L-2-chlorophenylalanin as internal standard was added to a 25 mg sample. The mixture was allowed to settle at −10 °C and treated via high throughput tissue crusher at 50 Hz for 6 min, then followed by ultrasound at 40 kHz for 30 min at 5 °C. The samples were placed at −20 °C for 30 min. After centrifugation at 13,000× *g* at 4° C for 15 min, the supernatant was transferred to sample vials for LC-MS/MS analysis by Shanghai Majorbio Bio-Pharm Technology Co., Ltd. (Shanghai, China). 

The LC-MS analytical platform was UHPLC-Q Exactive HF-X system (Thermo Fisher, USA). The reversed-phase separation was conducted with an ACQUITY UPLC HSS T3 column (100 mm × 2.1 mm i.d., 1.8 μm; Waters, Milford, CT, USA). The mobile phases consisted of 0.1% formic acid in water/acetonitrile (95:5, *v*/*v*) (solvent A) and 0.1% formic acid in acetonitrile/isopropanol/water (47.5:47.5:5, *v*/*v*) (solvent B). Gradient elution was performed as shown in Table S1. The sample injection volume was 3 μL and the flow rate was set to 0.4 mL/min. The column temperature was maintained at 40 °C. The mass spectrum signals were adopted by the positive (POS) and negative (NEG) ion scanning mode. The experimental conditions were optimized as follows: the heater temperature was set to 425 °C, the capillary temperature was maintained at 325 °C, the sheath gas flow rate was set to 50 arb, and the auxiliary gas flow rate was set to 13 arb. In the NEG mode, the ion-spray voltage floating (ISVF) was set to −3500 V, while in the POS mode, it was set to 3500 V. The normalized collision energy for MS/MS was varied at 20–40–60 V. The full MS resolution was set at 60,000, and the MS/MS resolution was set at 7500. Data acquisition was performed using the data-dependent acquisition (DDA) mode. The detection range spanned from 70 to 1050 *m*/*z*. A pooled quality control (QC) sample was prepared by mixing equal volumes of all samples. The QC samples were disposed and tested in the same manner as the analytic samples. To ensure the stability of the analysis during UPLC-MS, a QC sample was injected after every six analyzed samples.

### 2.5. Potential Metabolites Identification in C. fimbriata

The LC-MS raw data were processed using the metabolomic processing software Progenesis QI (Version 2.3, Waters Corporation, Milford, CT, USA). The preprocessing steps included baseline filtering, peak identification and integration, retention time correction, peak alignment, and normalization. To search and identify the potential metabolites, the main databases including the human metabolome database (HMDB) (http://www.hmdb.ca/, accessed on 1 February 2023), Metlin (https://metlin.scripps.edu/, accessed on 1 February 2023), and Majorbio Database were used. Then, the software was used to identify the characteristic peak database. MS and MS/MS information was matched with the metabolic database, MS mass error was set to be less than 10 ppm, and potential metabolites were identified according to the secondary mass spectrum matching score [33].

### 2.6. Potential Differentially Accumulated Metabolites (PDAMs) Analysis

After preprocessing the data, principal component analysis (PCA) and orthogonal partial least squares discriminant analysis (OPLS-DA) were performed to evaluate batch effects and detect outliers. To prevent overfitting of the model, a 7-cycle interactive validation approach was used. The significantly differentially accumulated metabolites were determined based on the variable importance (VIP) obtained by the OPLS-DA model and the *p*-value of student’s *t* test, and the metabolites with VIP > 1, *p* < 0.05 were PDAMs. PDAMs among two groups were summarized, and mapped into their biochemical pathways through metabolic enrichment and pathway analysis based on database search (KEGG, http://www.genome.jp/kegg/, accessed on 1 February 2023).

### 2.7. Statistical Analyses

The statistical analysis and data visualization were performed using GraphPad Prism 7.0 (GraphPad Software Inc., San Diego, CA, USA). The mean values are presented along with the standard deviation. To determine statistical differences between multiple groups, a one-way ANOVA with Tukey’s post hoc test was used. For comparisons between two experimental groups, a two-tailed Student’s *t* test was employed. Differences were considered significant if *p* < 0.05.

## 3. Results and Discussion

### 3.1. Deletion of CfSWI6 Resulted in Abnormal C. fimbriata Vegetative Growth

**Figure 1:**
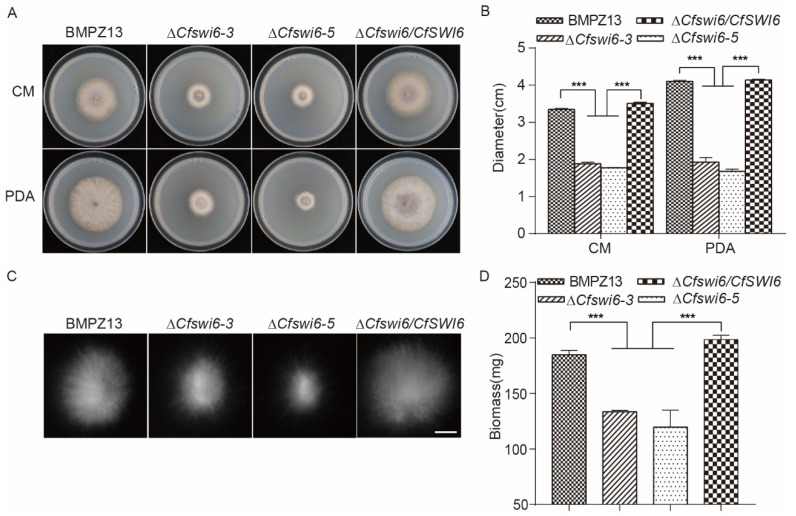
Vegetative growth of *C. fimbriata* and the mutants. (**A**) Colony growth of the tested strains on CM and PDA media at 27 °C for 5 days. (**B**) Colony diameter of the tested strains. (**C**) Hyphal balls of the tested strains cultured in CM liquid medium at 27 °C for 24 h (160 rpm). Images were taken using the M125C stereomicroscope (Leica, Wetzlar, Germany). Scale bar = 500 μm. (**D**) Biomass of the tested strains cultured in CM liquid medium at 27 °C for 3 days (160 rpm). Hyphae were separated via filtration and dried at 60 °C to a constant weight. The data were obtained from three independent experiments and are expressed as the mean ± SD. Multiple comparisons were conducted using a one-way ANOVA. Statistical significance was denoted as *** *p* < 0.001.

To assess the impact of *CfSWI6* on the growth and development of *C. fimbriata*, the vegetative growth of BMPZ13, ∆*Cfswi6* mutants, and the ∆*Cfswi6/CfSWI6* complemented mutant was examined on PDA and CM solid media (Figure 1A). The colony diameter of the ∆*Cfswi6* mutants exhibited a significant decrease on both media (Figure 1B). In the CM liquid medium, the hyphal balls of the ∆*Cfswi6* mutants were notably smaller compared to BMPZ13 (Figure 1C). Furthermore, the biomass of the ∆*Cfswi6-3* and ∆*Cfswi6-5* mutants cultured in CM liquid medium decreased by 27.7% and 35.3% compared with BMPZ13, respectively (Figure 1D). Conversely, these growth defects were absent in the ∆*Cfswi6/CfSWI6* complemented mutant. The findings suggested that *CfSWI6* played a critical role in the normal growth and development of *C. fimbriata*, aligning with previous studies that highlighted the involvement of APSES transcription factors in the regulation of fungal growth [21,22,23,29,34,35].

### 3.2. Deletion of CfSWI6 Gene Significantly Reduced the Virulence of C. fimbriata to Sweet Potato

**Figure 2:**
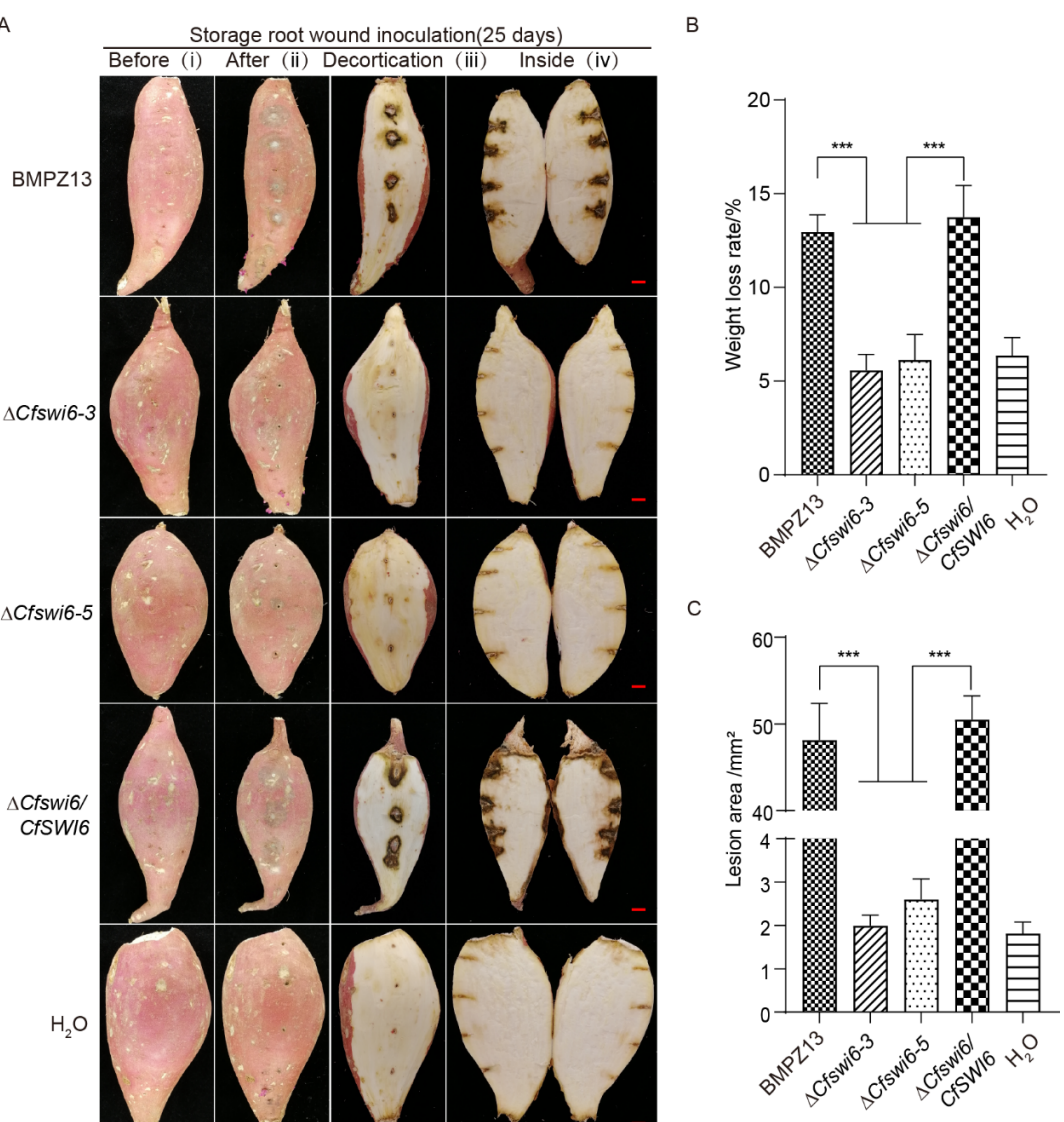
Virulence assays of *C. fimbriata* and the mutants to sweet potato. (**A**) Inoculation of wounded sweet potato storage roots with conidial suspensions of *C. fimbriata*. (**B**) Weight loss rate of sweet potato storage roots after wound inoculation with the tested strains for 25 days. (**C**) Lesion area of sweet potato storage roots after wound inoculation with the tested strains for 25 days. The data were obtained from three independent experiments and are expressed as the mean ± SD. Multiple comparisons were conducted using a one-way ANOVA. Statistical significance was denoted as *** *p* < 0.001.

To assess the effect of *CfSWI6* on the virulence of *C. fimbriata* to sweet potato, pathogenicity assays of sweet potato storage roots with conidial suspensions of BMPZ13 and ∆*Cfswi6* were conducted. After incubating sweet potato storage roots with a suspension of *C. fimbriata* conidia for 25 days, significant differences were observed between samples inoculated with the wild-type strain and the mutant strains (Figure 2A). Inoculation with the wild-type strain resulted in pronounced depressions at the site of sweet potato storage root wounds, accompanied by browning of the epidermis and the presence of conspicuous aerial hyphae ((ii) in Figure 2A). Upon removal of the sweet potato storage root epidermis, distinct black spots were observed at the inoculation site, indicating tissue collapse and the production of a foul odor ((iii) and (iv) in Figure 2A). These symptoms closely resembled those observed in sweet potato storage roots infected with *C. fimbriata* in previous research [5,7,36]. There were no significant differences observed in the sweet potato storage roots, both in terms of the outer skin and internal tissues, between the mutant strain-inoculated group and the H_2_O. The measurements of weight loss and lesion area in the samples also confirmed the above conclusion (Figure 2B,C). Additionally, the phenotype of sweet potato storage roots inoculated with the complemented strains resembled that of the wild-type samples, suggesting the significant involvement of the *SWI6* gene in the virulence of *C. fimbriata*. This finding aligns with previous functional studies on the *SWI6* gene in other fungi [23,26,27,28,29]. 

**Figure 3:**
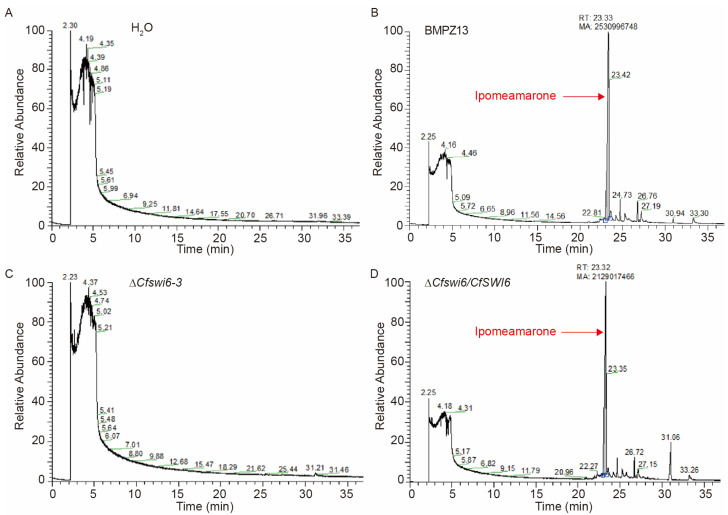
Detection profiles of ipomeamarone in sweet potato storage roots using GC-MS. (**A**–**D**). Quantification of ipomeamarone in sweet potato storage roots inoculated with H_2_O (**A**), BMPZ13 (**B**), ∆*Cfswi6*-3 (**C**), and the complemented strains Δ*Cfswi6/CfSWI6* (**D**) after 25 days. All inoculation assays were repeated three times.

In response to the attack by *C. fimbriata*, sweet potato employs a defense strategy by producing the toxins, including furanoterpenoids such as ipomeamarone [3,4,9,10]. Therefore, the content of ipomeamarone in sweet potato samples inoculated with *C. fimbriata* was determined using GC-MS analysis. GC-MS analysis of the total ion chromatogram revealed the presence of a compound with a profile suspected to ipomeamarone (by comparing with the structure of ipomeamarone in the gas chromatographic library) at a retention time of 23.3 min in sweet potato storage roots inoculated with the BMPZ13 and the complemented strains (Figure 3B,D). In contrast, this compound was not detected in the H_2_O or ∆*Cfswi6* mutant-inoculated sweet potato samples (Figure 3A,C). The results demonstrated the loss of pathogenicity of the ∆*Cfswi6* towards sweet potato storage roots.

### 3.3. Disruption of SWI6 Gene Altered the Potential Metabolite Profile of C. fimbriata

**Figure 4:**
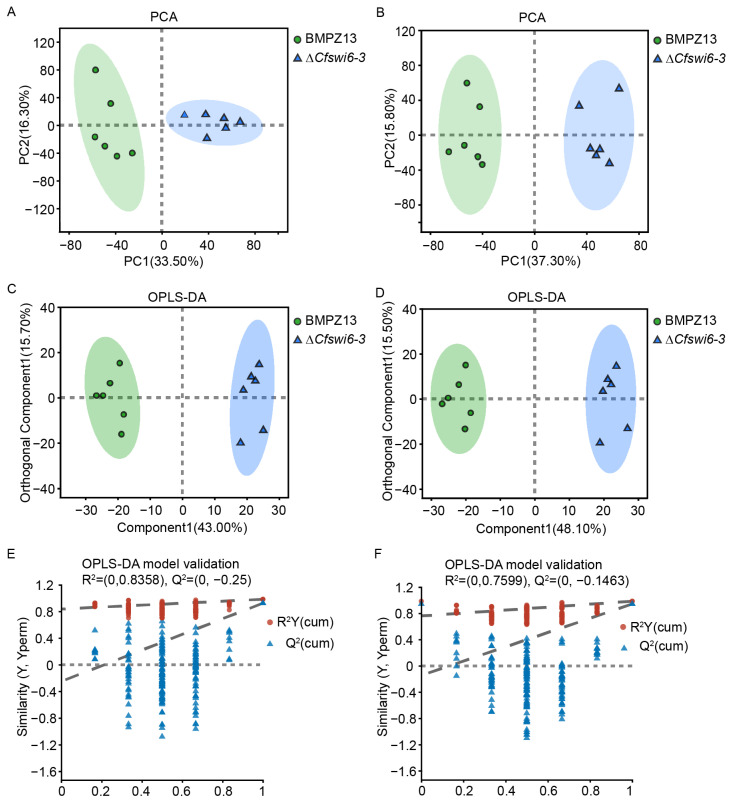
Quality control of non-targeted metabolomics data of *C. fimbriata* (BMPZ13 and ∆*Cfswi6-3*). (**A**,**B**) PCA score plots of BMPZ13 and ∆*Cfswi6-3* in POS (**A**) and NEG mode (**B**). (**C**–**F**) Validation of OPLS-DA models of pairwise comparation among BMPZ13 and ∆*Cfswi6-3* in POS (**C**,**D**), and NEG mode (**E**,**F**). In (**E**,**F**), the *x*-axis represents the retention of the permutation test, while the *y*-axis represents the values of the permutation test. R^2^Y (cum) represents the cumulative explained variance in the model for the Y matrix, and Q^2^ (cum) represents the predictive ability of the model. The closer these values are to 1, the more reliable the model is. The two dashed lines represent the regression lines for the explained variance and predicted values of the Y matrix. R^2^ and Q^2^ represent the intercept values of the regression lines with the *y*-axis.

PCA was conducted to evaluate the separation between the potential metabolite profiles of the BMPZ13 and ∆*Cfswi6-3* groups. The PCA score based on the first two principal components (PC) reached 49.8% (PC1 33.5%, PC2 16.3%) in POS mode (Figure 4A) and 53.1% (PC1 37.3%, PC2 15.8%) in NEG mode (Figure 4B), resulting in a cumulative difference of 60.8% and 64.4% (R^2^X (cum) in Table 1), and explaining the difference in the distribution of BMPZ13 and ∆*Cfswi6-3* groups. Notably, all six biological replicates fell within the 95% confidence circle of the sample, indicating a high level of similarity within the group and significant differences between the groups (Figure 4A,B). These findings suggested a substantial alteration in the potential metabolic profile of *C. fimbriata* following the deletion of the *CfSWI6* gene.

To further investigate the differences in the potential metabolic profile of *C. fimbriata* after *SWI6* gene deletion, we performed an OPLS-DA analysis (Figure 4C–F). The results revealed that the OPLS-DA model exhibited strong goodness of fit (R^2^Y) and high predictability (Q^2^) values of 0.983 and 0.925 (POS, Table 1), and 0.984 and 0.943 (NEG, Table 1), respectively (Figure 4C,D). Additionally, the OPLS-DA model was cross-validated using a permutation test. The intercept of Q^2^ fitting line in *Y*-axis in positive and negative ion mode (−0.25 in POS mode and −0.1463 in NEG mode) was less than 0 (Table 1 and Figure 4E,F), indicating that the model was not overfitted. In summary, the preprocessing analysis of the LC-MC detection data and the reliability of the model provide a solid foundation for further analysis of the potential metabolic profile differences between BMPZ13 and ∆*Cfswi6-3*.

**Figure 5:**
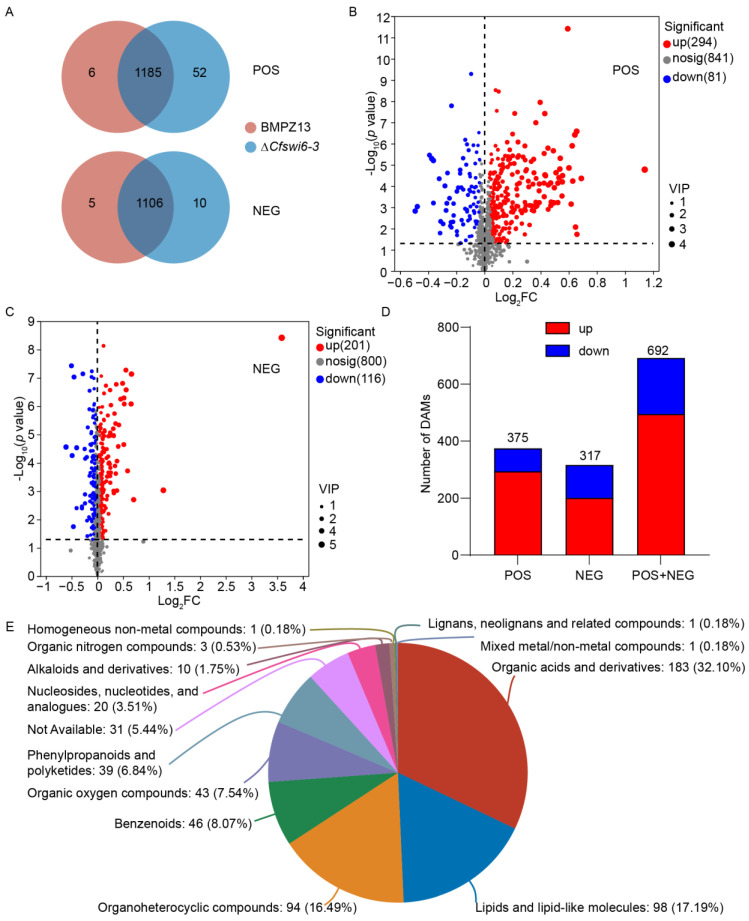
Analysis of potential metabolite quantity and composition in BMPZ13 and ∆*Cfswi6-3*. (**A**) Venn diagram of potential metabolites in BMPZ13, and ∆*Cfswi6-3* in POS and NEG modes. (**B**–**D**) Volcanic maps of PDAMs between wild type and mutants in POS (**B**) and NEG (**C**) modes and analysis (**D**). (**E**) The PDAMs classification maps based on the HMDB.

Based on the metabolomics database, the number of identified potential metabolites from the ion peaks detected by the mass spectrometer in both BMPZ13 and mutant strains is shown in Figure 5A. In the POS mode, a total of 1185 potential metabolites were detected in both samples, with 52 potential metabolites exclusively detected in the ∆*Cfswi6-3* and 6 metabolites specific to the BMPZ13 group (Table S2). In the NEG mode, the corresponding numbers of potential metabolites were 1106, 10, and 5, respectively.

Furthermore, using VIP > 1 and *p* < 0.05 as screening criteria, we identified 375 PDAMs (294 up-regulated and 81 down-regulated) in POS mode (Figure 5B and Table S4) and 317 PDAMs (201 up-regulated and 116 down-regulated) in NEG mode (Figure 5C, Table S5) when comparing wild-type and mutant samples. There were more PDAMs in POS mode when compared with the NEG mode (Figure 5D). Moreover, in both modes, the number of upregulated potential metabolites was significantly higher than the number of downregulated potential metabolites. These results indicated that the deletion of the *SWI6* gene led to an increased diversity of potential *C. fimbriata* metabolites, with a tendency for upregulation in the abundance of differentially expressed metabolites compared to BPMZ13. 

A total of 692 PDAMs were categorized into 13 categories (Table S6) based on the HMDB in Figure 5E. Among these metabolites, 183 PDAMs were classified as organic acids and derivatives, 98 PDAMs as lipids and lipid-like molecules, 94 PDAMs as organoheterocyclic compounds, 46 PDAMs as benzenoids, 43 PDAMs as organic oxygen compounds, 39 PDAMs as phenylpropanoids and polyketides, 20 PDAMs as nucleosides, nucleotides, and analogues, and 10 PDAMs as alkaloids and derivatives. Figure 5E illustrates that among the 13 selected categories, the majority of differentially expressed metabolites were found to accumulate in organic acids and derivatives, lipids, and lipid-like molecules, and organoheterocyclic compounds. This observation aligns with existing report that highlight the significance of these metabolite classes in microbial growth [37,38].

### 3.4. Hierarchical Cluster Analysis Revealed Potential Causes for Abnormal Growth and Pathogenicity in Mutant of C. fimbriata

**Figure 6:**
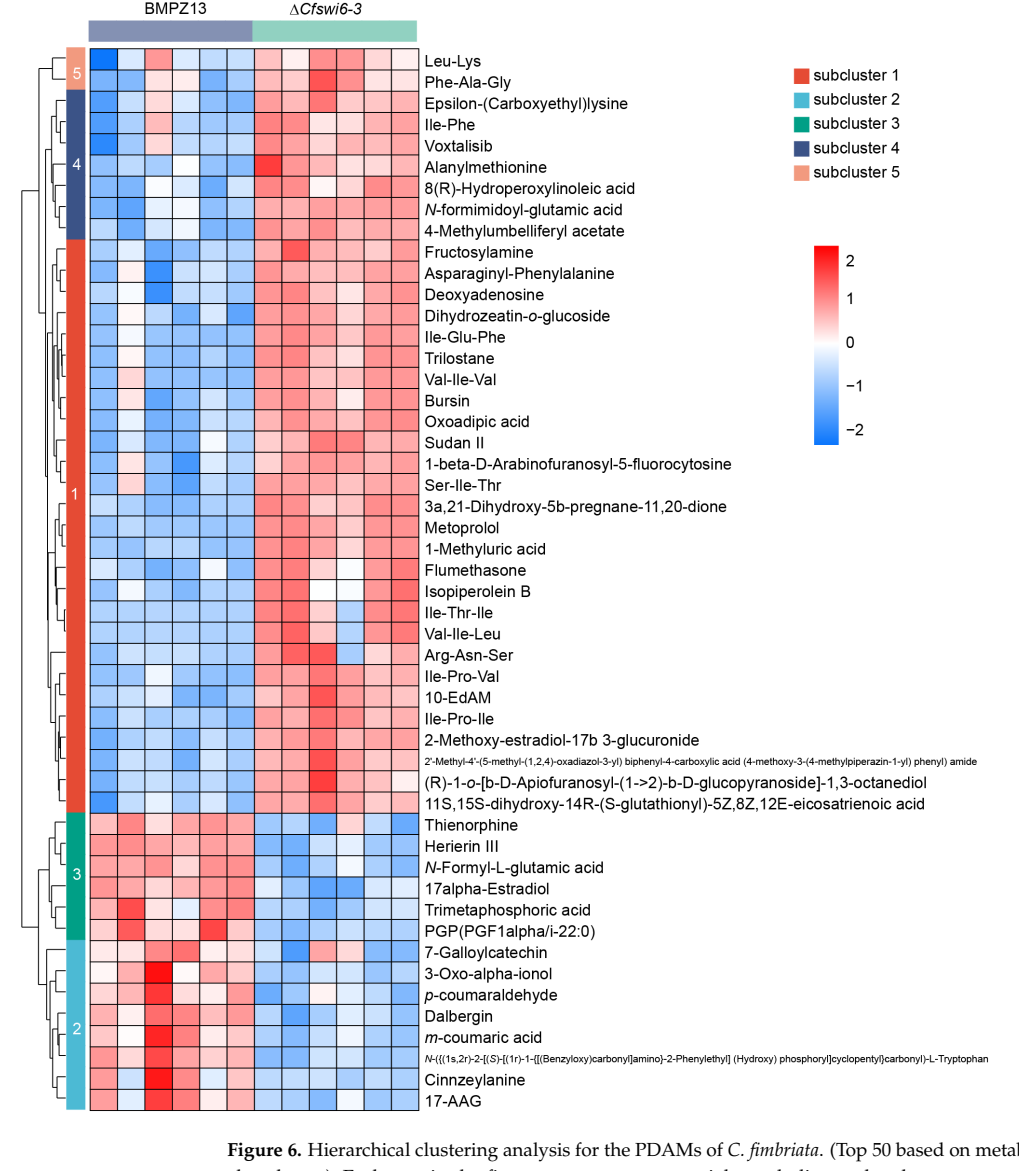
Hierarchical clustering analysis for the PDAMs of *C. fimbriata*. (Top 50 based on metabolic abundance.) Each row in the figure represents a potential metabolite, each column represents a sample, and the color represents the relative abundance of potential metabolites within the group. Red indicates high abundance levels, while blue indicates lower abundance levels.

To examine the distribution of differential metabolites within the potential metabolic profiles of wild-type and mutant strains, hierarchical cluster analysis was conducted on the top 50 PDAMs based on their abundance (Figure 6 and Table S7). Hierarchical clustering analysis revealed the formation of five distinct clusters, with the results indicating that the relative abundance of potential metabolites in clusters 1, 4, and 5 were upregulated in the mutant group compared to the wild-type group. Conversely, clusters 2 and 3 showed downregulation in the mutant group. Among the 36 upregulated potential metabolites (Table S7) of clusters 1, 4, and 5, 18 were classified as organic acids and derivatives, primarily amino acids, peptides, and analogues. Additionally, eight potential metabolites were classified as lipids and lipid-like molecules, while two metabolites belonged to nucleosides, nucleotides, and analogues. Studies have demonstrated the crucial role of amino acids in the metabolic processes of pathogenic fungi, as the balance of amino acid metabolism is essential for their successful host invasion [39,40,41]. In *M. oryzae*, the model species of filamentous fungi, disruption of lysine [42,43], leucine, isoleucine, and valine synthesis [41,44], as well as methionine [45,46] and arginine [40,47,48], significantly impacted mycelial development and virulence. Similarly, in human fungal pathogen *Cryptococcus neoformans*, the mutants without *ILV2* gene were unable to grow on nutrient-poor yeast nitrogen base (YNB) medium and exhibited loss of virulence [49]. Additionally, the ∆*ilv2* mutants in *Candida albicans* displayed a significant decrease in viability and a notable reduction in virulence during isoleucine and/or valine starvation [50]. Lipids and lipid-like molecules are essential components of cells and participate in various biological processes, including the formation of biological membranes, energy reserves, and signal transduction [51]. Studies have shown that abnormalities in lipid metabolism led to significant changes in fungal growth, development, and pathogenicity [52,53,54,55,56]. Compared to the wild type, the mutant exhibited a significant upregulation in the abundance of potential metabolites related to organic acids and derivatives, as well as lipids and lipid-like molecules. Among the 14 downregulated potential metabolites (Table S7), five were classified as phenylpropanoids and polyketides. Polyketide play a crucial role in fungal growth and pathogenicity. Melanin, a polyketide compound produced by *M. oryzae*, is essential for rice infection [29,57]. Pyriculol, a secondary metabolite of *M. oryzae*, induces rice leaf lesions, while extracts from *MoPKS19* (a polyketide synthase gene) knockout mutants fail to induce plant toxic lesions [58]. Nectriapyrone, a polyketide compound produced by various fungi, is involved in the growth and pigment formation of *Streptomyces griseus* [57]. This suggests that the deletion of the *SWI6* gene resulted in abnormal metabolic profiles in the mutant strain. This abnormality in metabolism may be a crucial factor contributing to the significant decrease in growth and virulence of the ∆*Cfswi6* strains towards sweet potato.

### 3.5. Impact of Potential Key Metabolites on Growth and Pathogenicity of C. fimbriata Based on VIP

**Figure 7:**
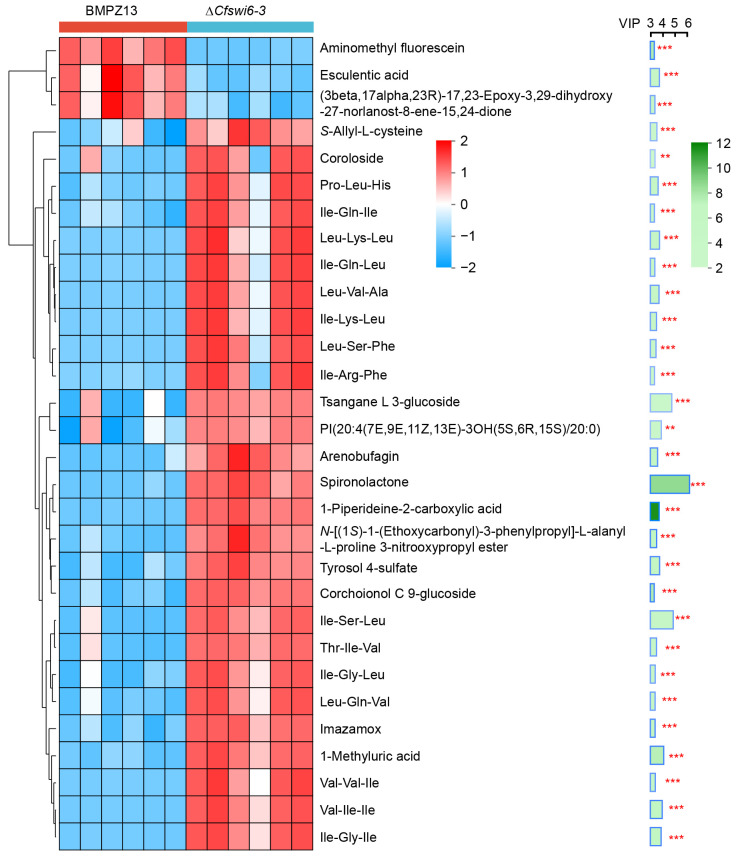
(Top 30) score chart of the PDAMs between BMPZ13 and ∆*Cfswi6-3* under OPLS-DA model. The figure on the left displays the potential metabolite clustering tree diagram, where the branches represent the similarity in abundance patterns of potential metabolites across samples. Each column represents a sample. Each row represents a potential metabolite, and the color indicates the relative abundance level of the metabolite within the sample group. Red indicates high abundance levels, while blue indicates lower abundance levels. The potential metabolites VIP bar chart is presented on the right side. The length of each bar represents the contribution value of the potential metabolite to the difference between the two groups being compared. A larger value indicates a greater difference between the groups. The green color in the bar chart represents the significance of the difference in potential metabolites between the two sample groups, as indicated by the *p*-value. A darker green color indicates a smaller *p*-value, indicating a more significant difference between the two groups. ** *p* < 0.01, *** *p* < 0.001.

To investigate the significance and abundance trends of PDAMs between the mutants and wild-type groups, we utilized the VIP score within the framework of the OPLS-DA model to evaluate PDAMs. The top 30 potential metabolites exhibiting significant differences in VIP score between the two groups were identified (Figure 7 and Table S8). According to Figure 7, significant differences were observed in the relative abundance of 30 potential metabolites between the two groups (*p* < 0.01). Notably, aminomethyl fluorescein, esculentic acid and (3beta,17alpha,23R)-17, 23-EPOxy-3, 29-Dihydroxy-27-Norlanost-8-ene-15,24-dione exhibited lower abundance levels in the mutant group compared to the wild type group. Conversely, the remaining potential metabolites displayed the opposite trend (Figure 7). The top 5 potential metabolites with the highest VIP scores were identified as Spironolactone, Ile-Ser-Leu, Tsangane L 3-glucoside, 1-Methyluric acid, and Val-Ile-Ile. Spironolactone is a steroid lactone, and existing research has demonstrated the impact of steroid hormones on fungal growth [59,60,61,62], morphology [63,64], and virulence [65,66]. Specifically, steroids disrupt fungal cell wall- or membrane-specific sensor proteins, leading to membrane stretching and activation of the CWI pathway [67,68]. Tsangane L 3-glucoside is a terpene glucoside, and previous studies have highlighted the essential role of glucosidases in carbohydrate metabolism, particularly in glycoprotein processing [69]. In fungi, defects in protein glycosylation pathways have been associated with reduced virulence, delayed dimorphism, and impaired interaction with the host immune system [70,71,72,73]. Furthermore, deficiencies in glucosidase activity led to changes in cell wall composition and tissue, ultimately activating the cell integrity pathway [74]. In *S. cerevisiae*, Swi6 was a transcription factor that functions downstream of the CWI-MAPK signaling pathway, playing a role in cell wall biogenesis and cell cycle regulation [68,75,76,77]. CWI defects resulting from the deletion of the *SWI6* gene have been observed in *Ganoderma lucidum* [35,78], *M*. *oryzae* [29], *B*. *bassiana* [23], *Fusarium granulosa* [28], and *Oligospora oligospora* [26]. Additionally, *SWI6* has been implicated in mediating morphological transformations in *B. bassiana* and *O. oligospora*, and these observations can be attributed to the involvement of *SWI6* in the regulation of the MAPK signaling pathway, which in turn mediates CWI [26,29,35,75,76,77,78,79,80]. Therefore, we speculate that the loss of the *SWI6* gene impairs the regulation of CWI in *C. fimbriata*, resulting in slow growth and decreased virulence.

Purines are fundamental constituents of nucleotides in living organisms, serving as essential metabolites for cellular physiology [81]. They play crucial roles as structural components of DNA and RNA, energy carriers, and enzyme cofactors [82]. Perturbations in purine nucleotide metabolism have been shown to significantly impact fungal development and virulence [81,82,83,84,85]. 1-Methyluric acid, a purine and purine derivative, has received limited attention in fungal research. However, in clinical studies on human kidney stones, abnormal purine metabolism has been associated with increased synthesis of 1-methyluric acid. This compound, a major component of caffeine metabolites, has been shown to induce innate immune cell responses and acute kidney injury. In mouse experiments, 1-methyluric acid affects the bladder mucosa, leading to elevated levels of serum glucose, insulin, true triglycerides, and total cholesterol, ultimately resulting in metabolic syndrome [86,87,88]. The observed higher abundance of 1-Methyluric acid in ∆*Cfswi6-3* compared to the wild type suggests a potential role for *SWI6* in regulating the growth and pathogenicity of *C. fimbriata* through purine nucleotide metabolism. 

### 3.6. SWI6 Mediated the Potential Growth and Virulence of C. fimbriata through Key Metabolic Pathways

**Figure 8:**
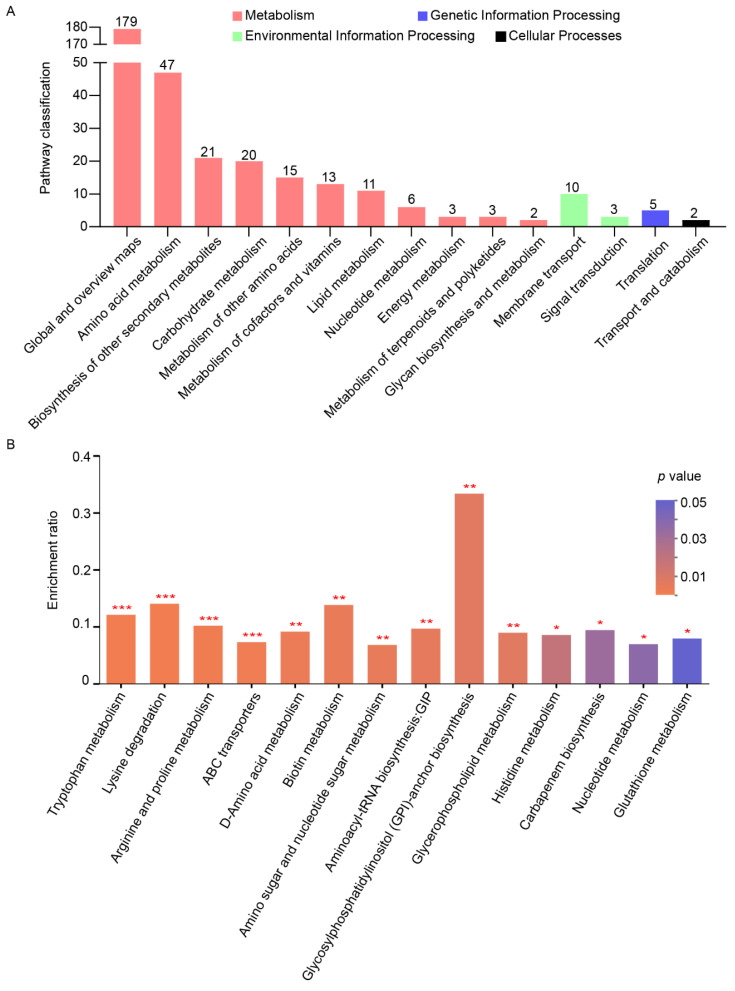
KEGG analysis of the PDAMs in the ∆*Cfswi6* compared with the BMPZ13. (**A**) KEGG pathway annotated and classification of the PDAMs. The *x*-axis represents level-2 terms of the KEGG pathway, and the *y*-axis represents the number of potential metabolites identified. (**B**) KEGG enrichment analysis of the PDAMs. The *x*-axis represents KEGG pathway, and the *y*-axis represents enrichment rate, which represents the ratio of potential metabolite number enriched in this pathway to the background number annotated in the pathway. The column color gradient represents the significance of enrichment. * *p* < 0.05, ** *p* < 0.01, *** *p* < 0.001.

To elucidate the potential key metabolic or biosynthetic pathways linked to the differential metabolites, annotation, and analysis of the 692 PDAMs were performed using the KEGG database, resulting in the classification into 15 KEGG second-grade pathways (Figure 8A and Table S9). Among these pathways, 320 potential metabolites were classified under “metabolism”. Within the “Metabolism” category, the top priority pathway was “Global and overview maps”, followed by “Amino acid metabolism”, “Biosynthesis of other secondary metabolites”, “Carbohydrate metabolism”, “Metabolism of other amino acids”, “Metabolism of cofactors and vitamins”, “Lipid metabolism”, “Nucleotide metabolism”, “Energy metabolism”, “Metabolism of terpenoids and polyketides”, and “Glycan biosynthesis and metabolism”. In addition, a metabolic pathway enrichment analysis was conducted on the 692 PDAMs (Figure 8B and Table S10). The results revealed significant differences in the enrichment rates of these potential metabolites in 14 metabolic pathways when comparing the mutant strain to the wild type. Notably, the pathways tryptophan metabolism, lysine degradation, arginine and proline metabolism, and ABC transporters exhibited highly significant differences (*p* < 0.001). 

Tryptophan is an indispensable amino acid for the biosynthesis of the growth hormone indole-3-acetic acid (IAA) in fungi, and it plays a critical role in fungal pathogenicity [89]. Treatment with tryptophan has been shown to enhance the production of 15-acetyldeoxynivalenolchanl in *F. graminearum* [90]. Moreover, in *Sporisorium scitamineum*, tryptophan-related synthetic enzymes exert a positive regulatory effect on filamentous growth, oxidative stress tolerance, and full pathogenicity [91]. Perturbations in lysine metabolism have been found to impede mycelial development and diminish virulence in *M. oryzae* [42,43] and *A. oligospora.* Arginine is a semi-essential amino acid crucial for spore germination, growth, and pathogenicity in fungi. Disruption of arginine biosynthesis genes leads to defects in asexual reproduction and pathogenicity in *Colletotrichum gloeosporioides* [92,93] and *M*. *oryzae* [40,47,48]. Arginine also plays a vital role in mycelial morphology through nitrogen metabolism in *C*. *albicans* [94], *Metarhizium acridum* [95], and *Arbuscular Mycorrhizal* [96]. Furthermore, arginine accumulation inhibits the synthesis of fungal toxins such as ochratoxin (OTA), citrinin (CIT), alternariol (AOH), alternariol monomethylether (AME), and tenuazonic acid (TeA) in various fungal species [97]. Proline acts as an antioxidant and osmoprotectant, effectively scavenging reactive oxygen species in *S. cerevisiae* cells and maintaining redox balance [98,99,100,101,102]. Furthermore, proline synthesis plays a crucial role in the fungal morphogenesis and pathogenicity of *C. albicans* [103,104]. The enrichment analysis of KEGG pathways suggests that disruption of *SWI6* perturbs the metabolism of tryptophan, lysine, arginine, and proline, which are crucial for fungal growth and development. This provides a potential reasonable explanation for the inability of the ∆*Cfswi6* to colonize sweet potato storage roots and exhibit abnormal pathogenicity.

ABC transporters, the largest gene family of transporter proteins, are present in all organisms [105]. They consist of structurally conserved nucleotide-binding domains and transmembrane domains [106]. By harnessing the energy derived from ATP hydrolysis, ABC transporters facilitate the transmembrane transport of diverse substrates, including amino acids, proteins, metal ions, fungal secretions, metabolites, sugars, peptides, and antibiotics [107]. Beyond their transport role, ABC transporters also participate in various physiological processes such as virulence, mitochondrial iron homeostasis, fatty acid metabolism, ribosome biogenesis, and RNA translation [106,108,109]. Notably, in plant pathogenic fungi like *M. oryzae* [110,111], *F. graminearum* [106,112,113,114], *Neofusicoccum parvum* [115], *Mycosphaerella graminicola* [116,117], *Colletotrichum lindemuthianum* [118], *C. albicans* [119,120], and *Botrytis cinerea* [121], ABC transporters have been identified as an important virulence determinant. In Figure 8B, the deletion of *SWI6* in *C. fimbriata* leads to a significant enrichment of ABC transporters in the metabolic profile. This observation implies that ABC transporters potentially have a crucial involvement in the virulence of *C. fimbriata*, primarily through their transport function.

Among these pathways, Glycosylphosphatidylinositol-anchor biosynthesis displayed the highest enrichment rate (Figure 8B). Glycosylphosphatidylinositol (GPI) is a conserved complex glycolipid derived from the endoplasmic reticulum (ER) in eukaryotes [122,123]. It acts as a cell surface anchor for over 100 cell wall proteins in yeast, initially covalently linked to β1-6 glucan [124,125]. Previous research has revealed that manogepix, a novel antifungal drug, targets the maturation of GPI-anchored proteins by inhibiting Gwt1, an inositol acyltransferase in the ER [126]. Gwt1 is essential for the transport and anchoring of mannose-containing proteins to the fungal cell membrane and wall [127]. Furthermore, the inability of GPI proteins to anchor to the plasma membrane or successfully crosslink with β-1,6-glucan polymers may fail to form glycosylphosphatidylinositol-cell wall proteins (GPI-CWPs) [128], which constitute the outer layer structure of the cell wall, thereby disrupting cell wall integrity. Thus, the biosynthesis and metabolism of GPI-anchored proteins were vital for fungal virulence and CWI [124,125,126,128,129,130,131,132,133]. Cell wall damage, stress sensitivity, and reduced pathogenicity in *SWI6* mutants observed in several fungi [26,28,29,78], may be attributed to abnormal GPI synthesis and metabolism. 

**Figure 9:**
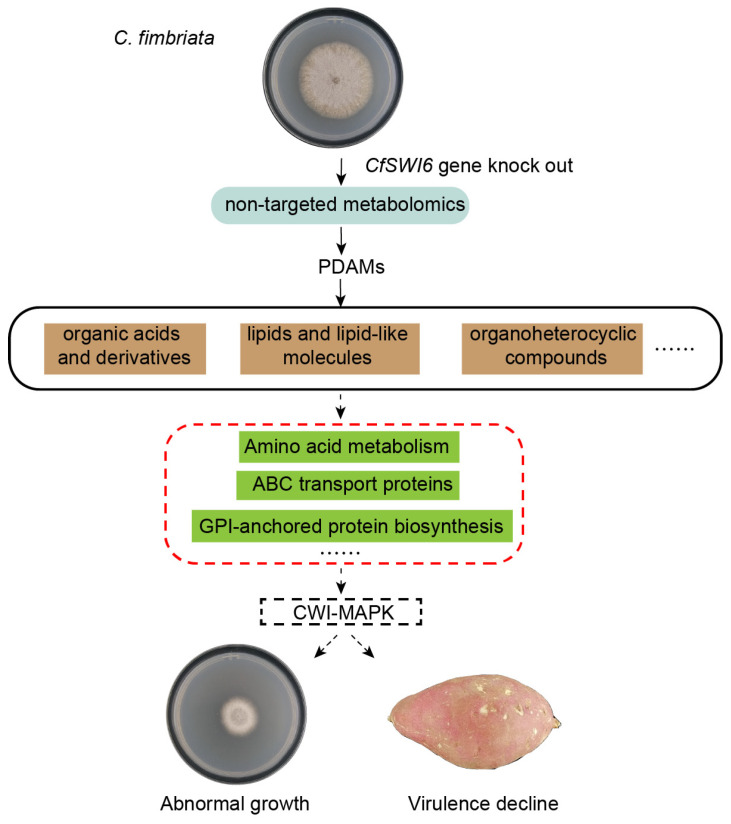
Schematic representation for the potential regulation of CfSwi6 in *C. fimbriata*.

Disruption of the *SWI6* gene in *C. fimbriata* affects potential key metabolic pathways, including tryptophan, lysine, arginine, and proline metabolism. It also impacts the function of ABC transporters, crucial for virulence. Additionally, this disruption affects GPI anchor biosynthesis, essential for cell wall integrity and fungal virulence. These factors likely form an interconnected regulatory network that collectively mediates the growth and pathogenicity of *SWI6* in sweet potato (Figure 9).

## 4. Conclusions

In this study, we investigated the essential factors contributing to the growth and virulence of *C. fimbriata* by characterizing the potential metabolic profile of the *SWI6* deletion mutants using LC-MS-based non-targeted metabolomics. Our findings demonstrated that the mutant strain exhibited differential abundance of potential metabolites, primarily belonging to organic acids and derivatives, lipids, and lipid-like molecules, and organoheterocyclic compounds, compared to the wild type. KEGG pathway enrichment analysis revealed significant enrichment in amino acid metabolism, ABC transport proteins, and GPI-anchored protein biosynthesis. These potential metabolic pathways are likely involved in mediating the virulence of *C. fimbriata* through processes such as energy supply, intracellular transmembrane transport, and cell wall integrity. Targeted identification and quantification of PDAMs identified in the analysis will be the focus of future investigations. Future studies also should evaluate the additional functions of the APSES transcription factor CfSwi6, such as its role in CWI and molecular mechanisms of transcriptional regulation of downstream target genes. These studies will provide valuable targets for the control of *C. fimbriata* in sweet potatoes, thereby enhancing the strategies for the biological management of sweet potato black rot disease.

## Figures and Tables

**Table 1:** Comparative analysis parameters for POS and NEG mode.

Ion Mode	PCA	OPLS-DA			
	R^2^X (cum)	R^2^Y (cum)	Q^2^ (cum)	R^2^	Q^2^
POS	0.608	0.983	0.925	0.8358	−0.25
NEG	0.644	0.984	0.943	0.7599	−0.1463

R^2^X (cum), the cumulative explanation rate for differences in variables in the PCA model.

## Data Availability

The non-targeted metabolomic data utilized in this study have been deposited in the EMBL-EBI MetaboLights database under the identifier MTBLS8267. The data have been successfully submitted and will be accessible through the following link upon completion of the database review: https://www.ebi.ac.uk/metabolights/MTBLS8267, accessed on 25 July 2023).

## Supplementary Materials

The following supporting information can be downloaded at: https://www.mdpi.com/article/10.3390/microorganisms11112666/s1, Figure S1: Targeted *CfSWI6* deletion and complementation in *C. fimbriata*; Table S1: Mobile phase elution gradient in LC-MS; Table S2: Potential metabolites in POS mode; Table S3: Potential metabolites in NEG mode; Table S4: PDAMs in POS mode; Table S5: PDAMs in NEG mode; Table S6: 13 categories of potential metabolites based on the HMDB; Table S7: Top 50 metabolites based on abundance in PDAMs; Table S8: Top 30 PDAMs in VIP score; Table S9: KEGG pathway annotated and classification of the PDAMs; Table S10: KEGG enrichment analysis of the PDAMs.
