# Supplementary material for: The Mechanism of Transcription Factor Swi6 in Regulating Growth and Pathogenicity of Ceratocystis fimbriata: Insights from Non-Targeted Metabolomics
Source: Microorganisms. 2023 Oct 30;11(11):2666. doi: 10.3390/microorganisms11112666 (PMC10673406; doi:10.3390/microorganisms11112666)
Supplement: Supplementary file 1 [file microorganisms-11-02666-s001.zip › Supplementary Figure S1/Figure S1 and legend.pdf]

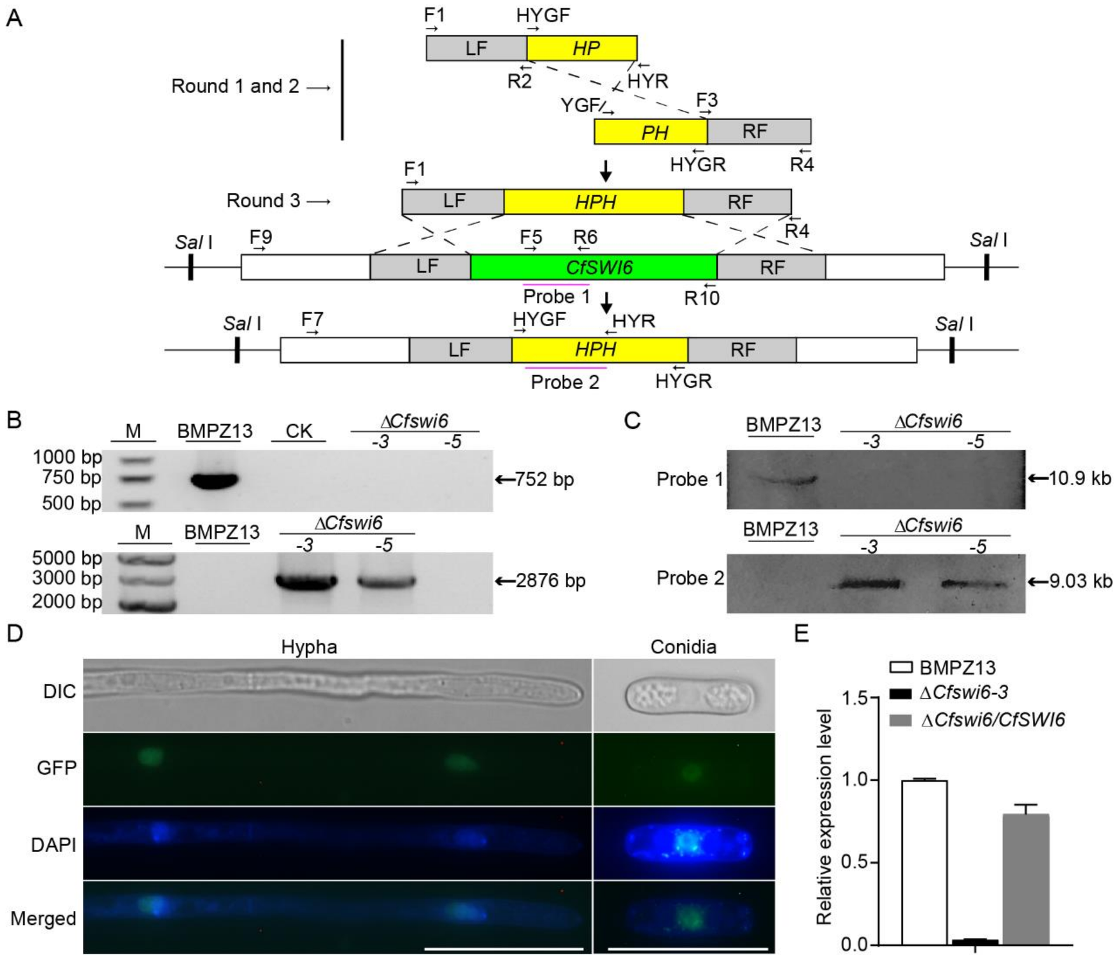

Figure S1: Targeted *CfSWI6* deletion and complementation in *C. fimbriata*. (A) Schematic representation of the targeted deletion and complementation of *CfSWI6*. Disruption of the *CfSWI6* sequence was achieved by inserting the hygromycin phosphotransferase (*HPH*) resistance gene through three rounds of overlapping PCR. The orientations and positions of the primers for the overlapping PCR are labeled with small arrows, and all PCR primers used are listed in the table below. LF and RF refer to the upper and lower arms of *CfSWI6*, respectively. *HP* and *PH* refer to the forward and reverse parts of the *HPH* gene in the pCX62 plasmid. The knockout vector fragment was purified and used for transformation of *C. fimbriata* via the PEG-mediated protoplast method to gain the mutants. The complementary fragment containing the *CfSWI6* gene (no stop codon) and its upstream promoter region was amplified by PCR using *C. fimbriata* genomic DNA as the template and primers F9 and R10. The complementary fragment was then cloned into plasmid pYF11 using the yeast gap repair method, resulting in the DNA fragment pYF11-*CfSWI6*-GFP. To obtain the complemented mutant, protoplast transformation of the Δ*Cfswi6-3* mutant strains was performed using the pYF11-Cf*SWI6*-GFP plasmid, following the described method. (B) PCR validation of the *CfSWI6* deletion in the transformed mutants. Δ*Cfswi6-3* and Δ*Cfswi6-5* were validated by the internal (no band at 752 bp) and external (a band at 2,876 bp) PCR amplifications. The *C. fimbriata* DNA and CK (H_2O) were used as controls. (C) Southern blot analysis of the gene knockout mutants. The genomic DNA samples from *C. fimbriata* and the mutants were digested with *Sal* I and separated on a 1% agarose gel. The DNA samples from *C. fimbriata* and the mutants were hybridized with probe 1 and 2, respectively. A band at 10.9 kb was observed after the hybridization between the

DNA from *C. fimbriata* and probe 1. A band at 9.03 kb was observed after the hybridization between the DNA from the mutants and probe 2. The DiG High Prime DNA Labeling and Detection Starter Kit 1 (Roche, Germany) was used in Southern blot analysis. (D) The expression and subcellular localization of CfSwi6-GFP in Δ*Cfswi6/CfSWI6* complemented strains. The Δ*Cfswi6/CfSWI6* complemented mutant was obtained by transforming Δ*Cfswi6-3*. The Δ*Cfswi6/CfSWI6* hyphae and conidia were stained with 4,6-diamino-2-phenylindole (DAPI) and then examined using the DM5000B fluorescence microscope (Leica, Germany). The green fluorescence channel revealed the localization of CfSwi6-GFP. The location of the nucleus was indicated by the DAPI stain. Captured images of the DAPI (blue) and GFP (green) fluorescence as well as the merged image processed using the Photoshop software are presented. Scale bar = 20 μm. (E) The relative expression levels of the *SWI6* gene in the mutant strains and complemented strains. Total RNA of the wild-type, Δ*Cfswi6-3* and Δ*Cfswi6/CfSWI6* complemented mutants were extracted using the Fungal Total RNA Isolation Kit (Sangon Biotech, China)， and reverse transcribed to cDNA using the HiScript II Q RT Supermix for qPCR (Vazym Biotech, China). The quantitative real-time PCR (qRT-PCR) was performed using the ChamQ SYBR qPCR Master Mix (Vazym Biotech, China) and specific primer (in the table below) with an StepOne Plus real-time PCR System (ABI, United States). The relative transcript levels were calculated using the $2^{-\Delta\Delta Ct}$ method, and the average cycle threshold (Ct) was normalized to that of β -Actin. The cycling program was as follows: 95℃ for 30 s, followed by 40 cycles of 95℃ for 10 s and 60℃ for 30 s. All qRT-PCR assays were performed in triplicate and repeated three times.

Table. Paired primers used for *CfSWI6* knockout and verification

Primer name/ Purpose	Primer sequence (5'-3')
F1	GCTAAGGGCAAAAGACACGC
R2	TTGACCTCCACTAGCTCCAGCCAAGCCTAGCTGGTGGCGATGATACG
F3	CAAAGGAATAGAGTAGATGCCGACCGTCTTTCTCTTGCGCTCGTCT
R4	GTATGCCATGGGTGAGGCTT
F5	CTCAGCCTTAGTAGCGCCTC
R6	TCGAGAATCTTGGTGCGCTT
F7	ACAGAACGCTGCTGATCCAT
F9	ACTCACTATAGGGCGAATTGGGTACTCAAATTGGTTACAGAACGCTGCTGATCCAT
R10	CACCACCCCGGTGAACAGCTCCTCGCCCTTGCTCACTCGCACAGCGCCTTCCTCA
HYGF	GGCTTGGCTGGAGCTAGTGGAGGTCAA
HYR	TATTGACCGATTCCTTGCGGTCCGAA
YGF	GATGTAGGAGGGCGTGGATATGTCCT
HYGR	CGGTCGGCATCTACTCTATTCCTTTG
First round of overlap PCR	(F1, R2); (F3, R4); (HYGF, HYR); (YGF, HYGR)
Second round of overlap PCR	(F1, HYR); (YGF, R4)
Third round of overlap PCR	(F1, R4)
Complementary fragment	(F9, R10)
Internal verification of PCR	(F5, R6)
External verification of PCR	(F7, HYGR)
Probe 1 of Southern blot	(F5, R6)
Probe 2 of Southern blot	(HYGF, HYR)
Paired primers of β -Actin for qRT-PCR	GTCACTCACGTCGTTCCCAT; CACGCTCGGCAGTAGTAGAG
Paired primers of *SWI6* for qRT-PCR	CACGAAAAGGACTAGCACCA; AGCATCAAGGGCATGACACC
