# Supplementary material for: The Mechanism of Transcription Factor Swi6 in Regulating Growth and Pathogenicity of Ceratocystis fimbriata: Insights from Non-Targeted Metabolomics
Source: Microorganisms. 2023 Oct 30;11(11):2666. doi: 10.3390/microorganisms11112666 (PMC10673406; doi:10.3390/microorganisms11112666)
Supplement: Supplementary file 1 [file microorganisms-11-02666-s001.zip › Supplementary Figure S1/The gel and blot results .pdf]

The gel and blot results in Figure S1

1、Figure S1 B: PCR validation of the *CfSWI6* deletion in the transformed mutants.

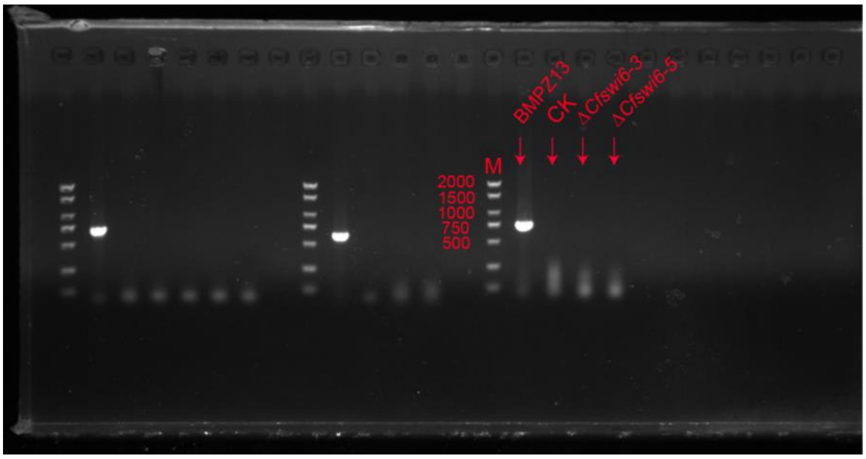

Note: This is the original image of the internal PCR validation (five electrophoresis lanes with labels on the far right). *C. fimbriata* (BMPZ13) DNA and CK (H_2O) were used as positive and negative controls, respectively. PCR analysis using primers F5 and R6 revealed that the Δ*Cfswi6-3* and Δ*Cfswi6-5* lanes showed no bands, while the BMPZ13 lane exhibited a band of size 752 bp. The image was cropped and inversion processed to get Figure S1 b.

2、Figure S1 B: PCR validation of the *CfSWI6* deletion in the transformed mutants.

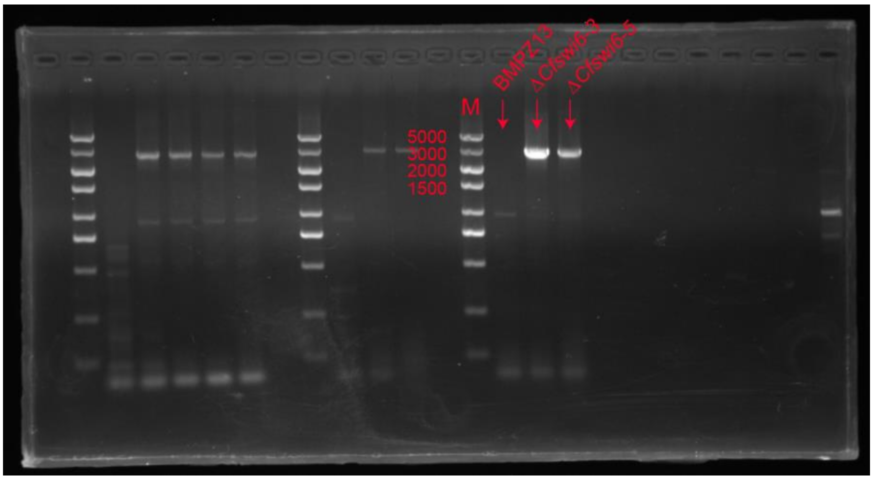

Note: This is the original image of the external PCR validation (four electrophoresis lanes with labels on the far right). *C. fimbriata* (BMPZ13) DNA was used as negative controls. PCR analysis using primers F7 and HYGR revealed that Δ*Cfswi6-3* and Δ*Cfswi6-5* exhibited bands of size 2876 bp, while BMPZ13 did not show any bands. Figure S1 b was generated by clipping and inverting this image.

3、Figure S1 C:Southern blot analysis of the gene knockout mutants.

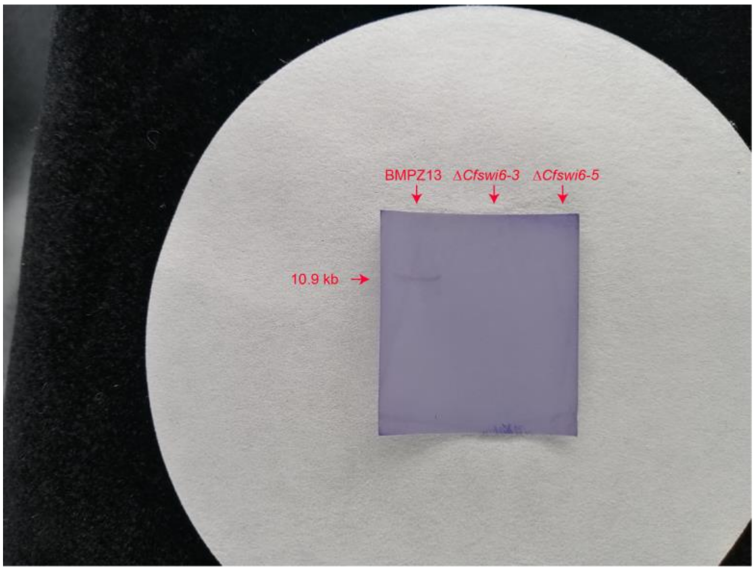

Note: This is the original image of probe 1 in Southern blot. Probe 1 exhibited specific hybridization with the DNA of *C. fimbriata*, while no hybridization was observed with the DNA of the mutants. The Lane Marker was removed after electrophoresis, and the nylon membrane was cut into two pieces before hybridization and reacted with the corresponding probe respectively. Figure S1c was obtained by cropping this image and adjusting the brightness and contrast.

4、Figure S1 C:Southern blot analysis of the gene knockout mutants.

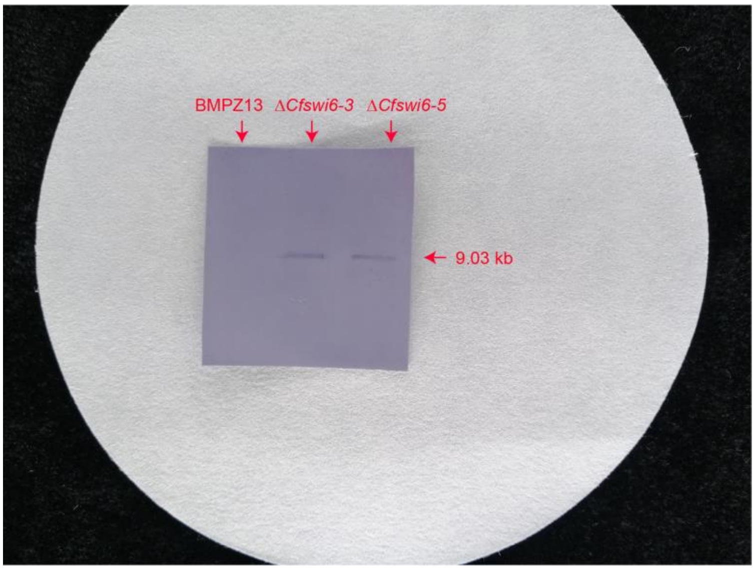

Note: This is the original image of probe 2 in Southern blot. Probe 2 exhibited specific hybridization with the DNA of the mutants, while not with *C. fimbriata*. The Lane Marker was removed after electrophoresis, and the nylon membrane was cut into two pieces before hybridization and reacted with the corresponding probe respectively. Figure S1c was obtained by cropping this image and adjusting the brightness and contrast.

5、Figure S1 D: The expression and subcellular localization of CfSwi6-GFP in ∆*Cfswi6/CfSWI6* complemented strains.

Hypha

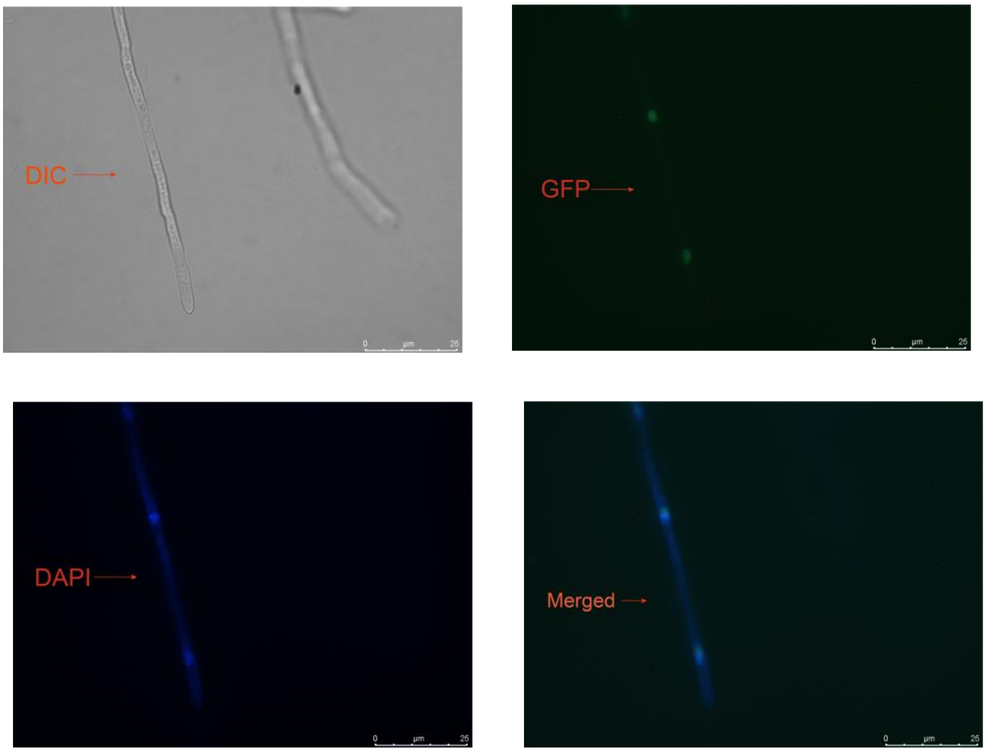

Conidia

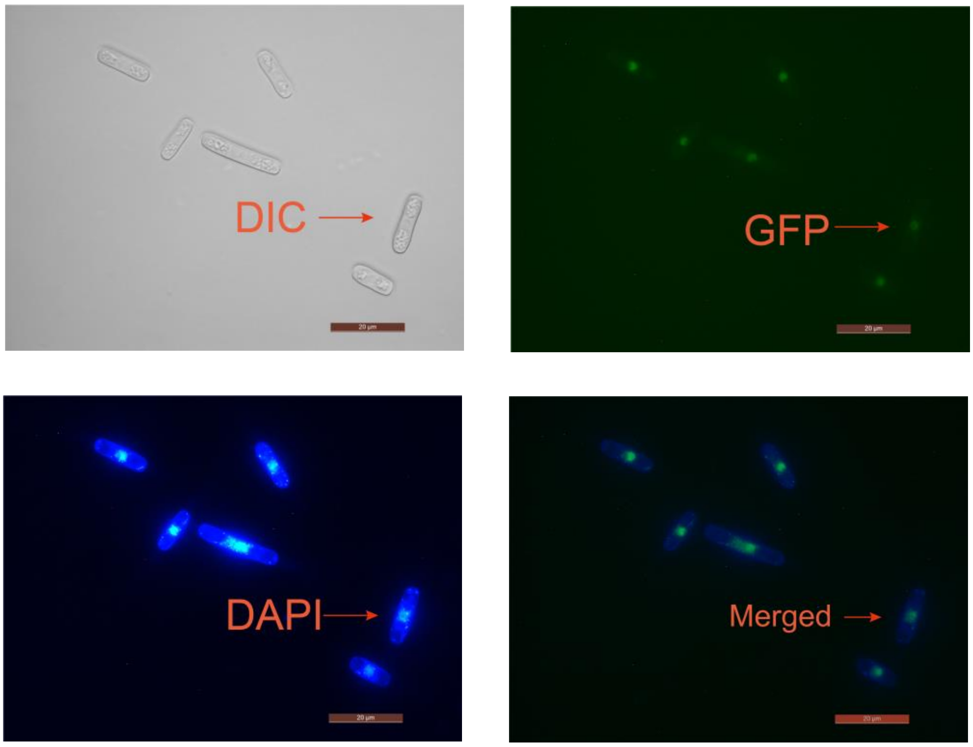

Note: This is the original image of Figure S1 D. The ∆*Cfswi6/CfSWI6* hyphae and conidia were stained with 4,6-diamino-2-phenylindole (DAPI) and then examined using the DM5000B fluorescence microscope (Leica, Germany). The green fluorescence channel revealed the localization of CfSwi6-GFP. The location of the nucleus was indicated by the DAPI stain. Captured images of the DAPI (blue) and GFP (green) fluorescence as well as the merged image processed using the Photoshop software are presented. Figure S1D was obtained by cropping this image and adjusting the brightness and contrast.
