# Supplementary material for: The Mechanism of Transcription Factor Swi6 in Regulating Growth and Pathogenicity of Ceratocystis fimbriata: Insights from Non-Targeted Metabolomics
Source: Microorganisms. 2023 Oct 30;11(11):2666. doi: 10.3390/microorganisms11112666 (PMC10673406; doi:10.3390/microorganisms11112666)
Supplement: Supplementary file 1 [file microorganisms-11-02666-s001.zip › Supplementary Table/Table S1 Mobile phase elution gradient in LC-MS.pdf]

Table S1. Mobile phase elution gradient in LC-MS

Table S1a. Mobile phase elution gradient in positive ion mode

Time （min）	Flow rate （mL/min）	A （%）	B （%）
0	0.4	100	0
3	0.4	80	20
4.5	0.4	65	35
5	0.4	0	100
6.3	0.4	0	100
6.4	0.4	100	0
8	0.4	100	0

Table S1b. Mobile phase elution gradient in negative ion mode

Time （min）	Flow rate （mL/min）	A （%）	B （%）
0	0.4	100	0
1.5	0.4	95	5
2	0.4	90	10
4.5	0.4	70	30
5	0.4	0	100
6.3	0.4	0	100
6.4	0.4	100	0
8	0.4	100	0

Note: Mobile phase A consists of 95% water + 5% acetonitrile (containing 0.1% formic acid), while mobile phase B consists of 47.5% acetonitrile + 47.5% isopropanol + 5% water (containing 0.1% formic acid).
