# Supplementary figures and images for: The Mechanism of Transcription Factor Swi6 in Regulating Growth and Pathogenicity of Ceratocystis fimbriata: Insights from Non-Targeted Metabolomics
Source: Microorganisms. 2023 Oct 30;11(11):2666. doi: 10.3390/microorganisms11112666 (PMC10673406; doi:10.3390/microorganisms11112666)

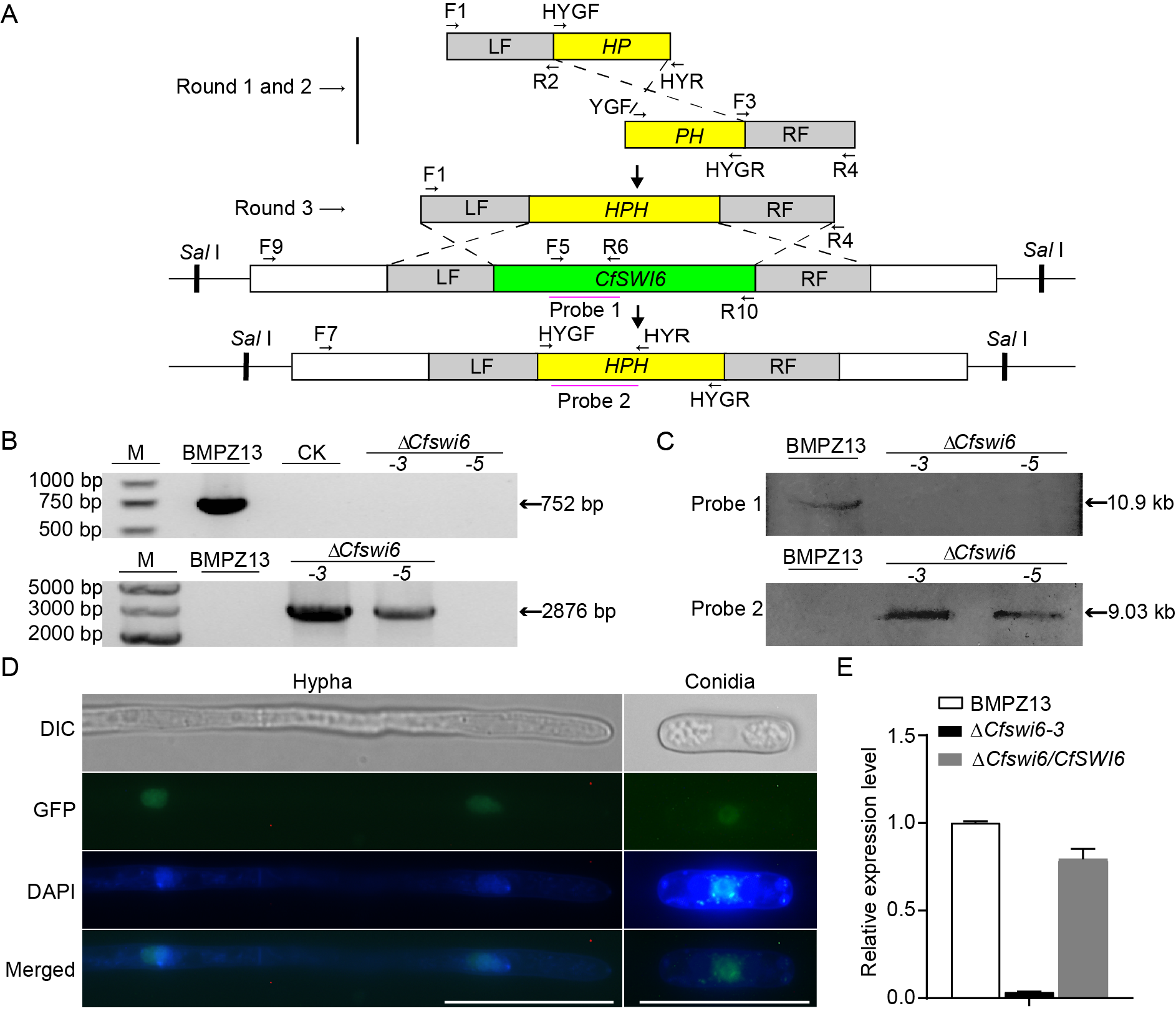

Supplement: Supplementary file 1 [file microorganisms-11-02666-s001.zip › Supplementary Figure S1/Figure S1.tif]

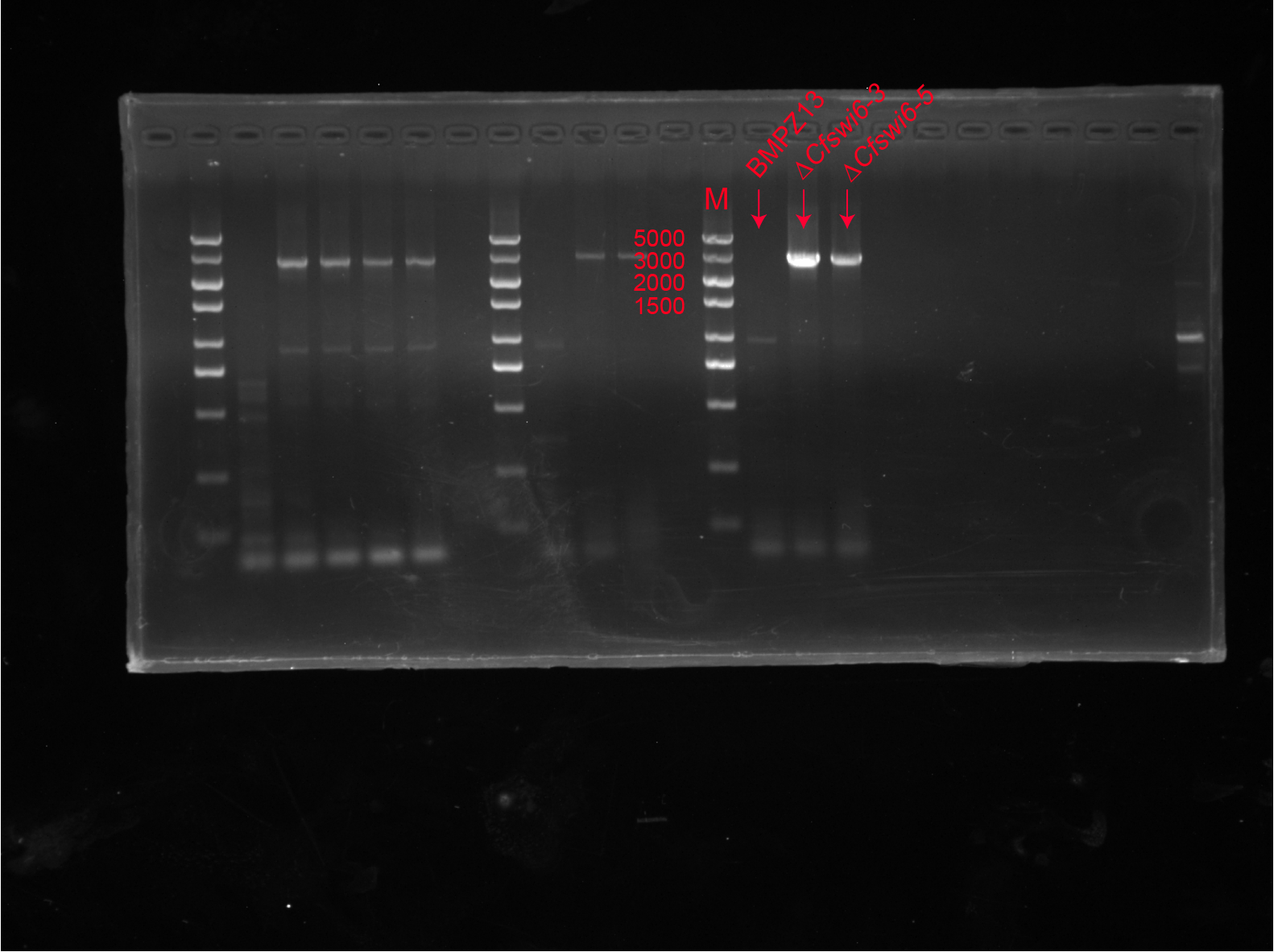

Supplement: Supplementary file 1 [file microorganisms-11-02666-s001.zip › Supplementary Figure S1/Figure S1B/Figure S1 B External PCR amplifications.tif]

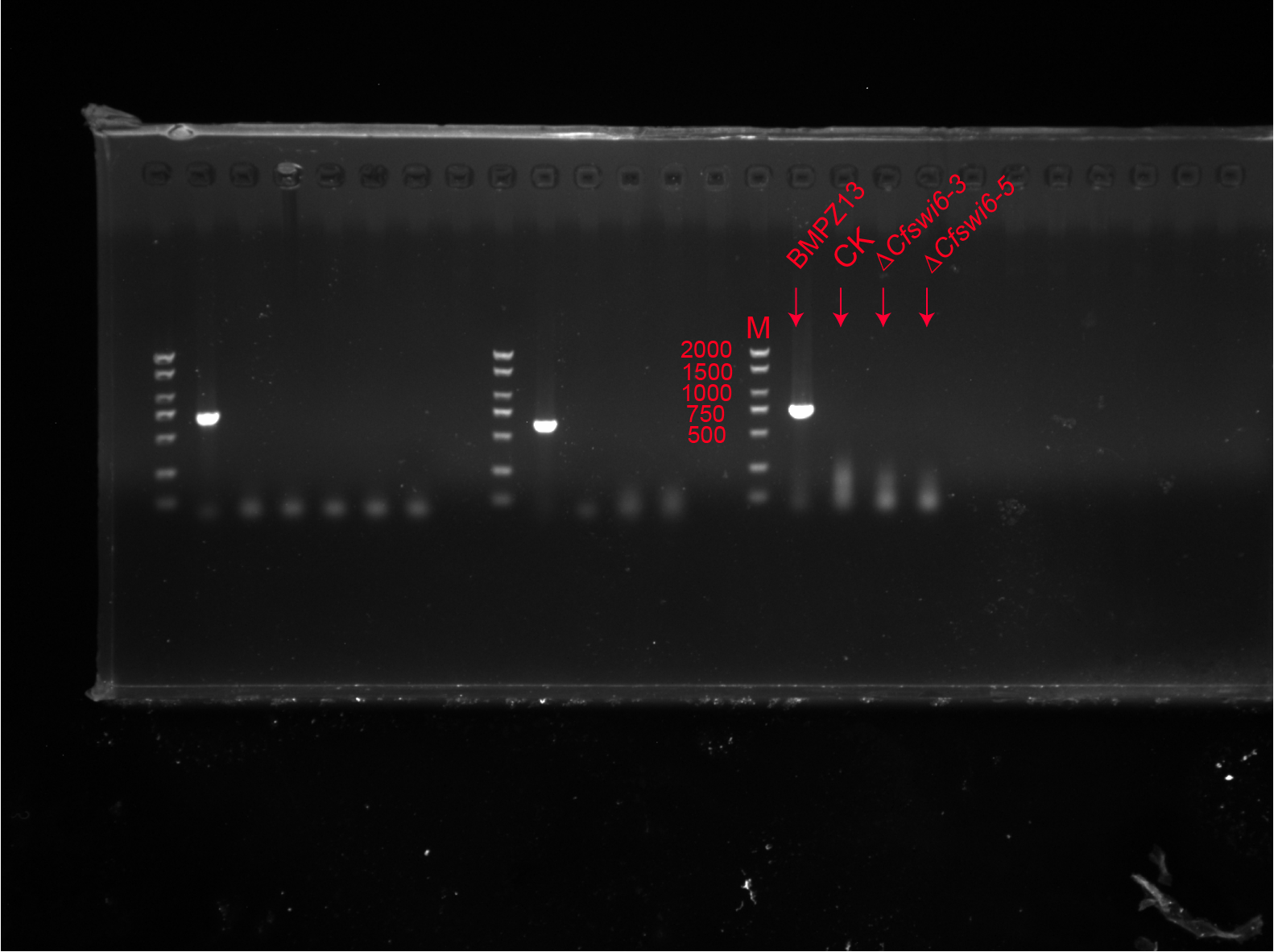

Supplement: Supplementary file 1 [file microorganisms-11-02666-s001.zip › Supplementary Figure S1/Figure S1B/Figure S1 B Internal PCR amplifications.tif]

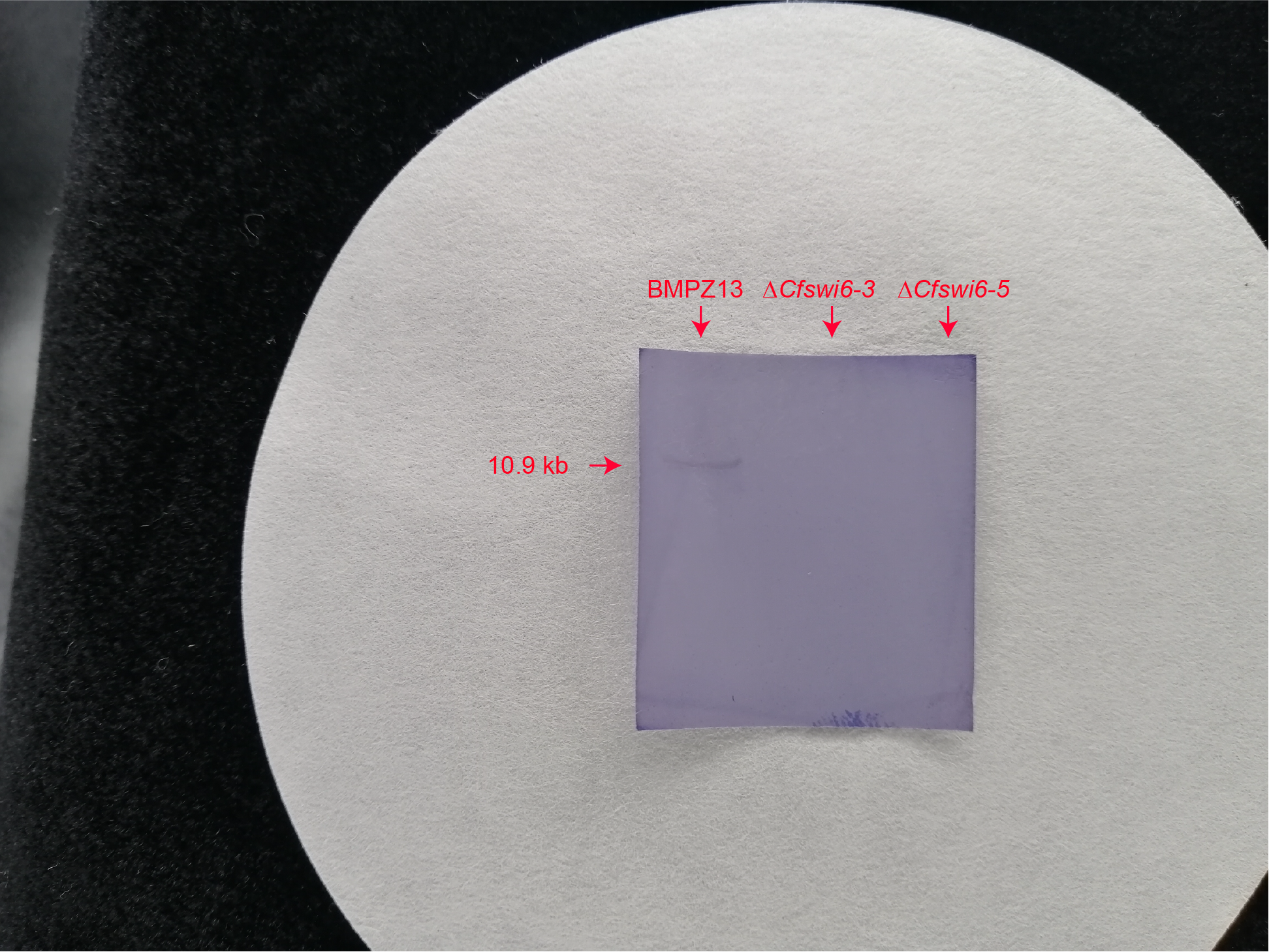

Supplement: Supplementary file 1 [file microorganisms-11-02666-s001.zip › Supplementary Figure S1/Figure S1C/Figure S1C Probe 1.tif]

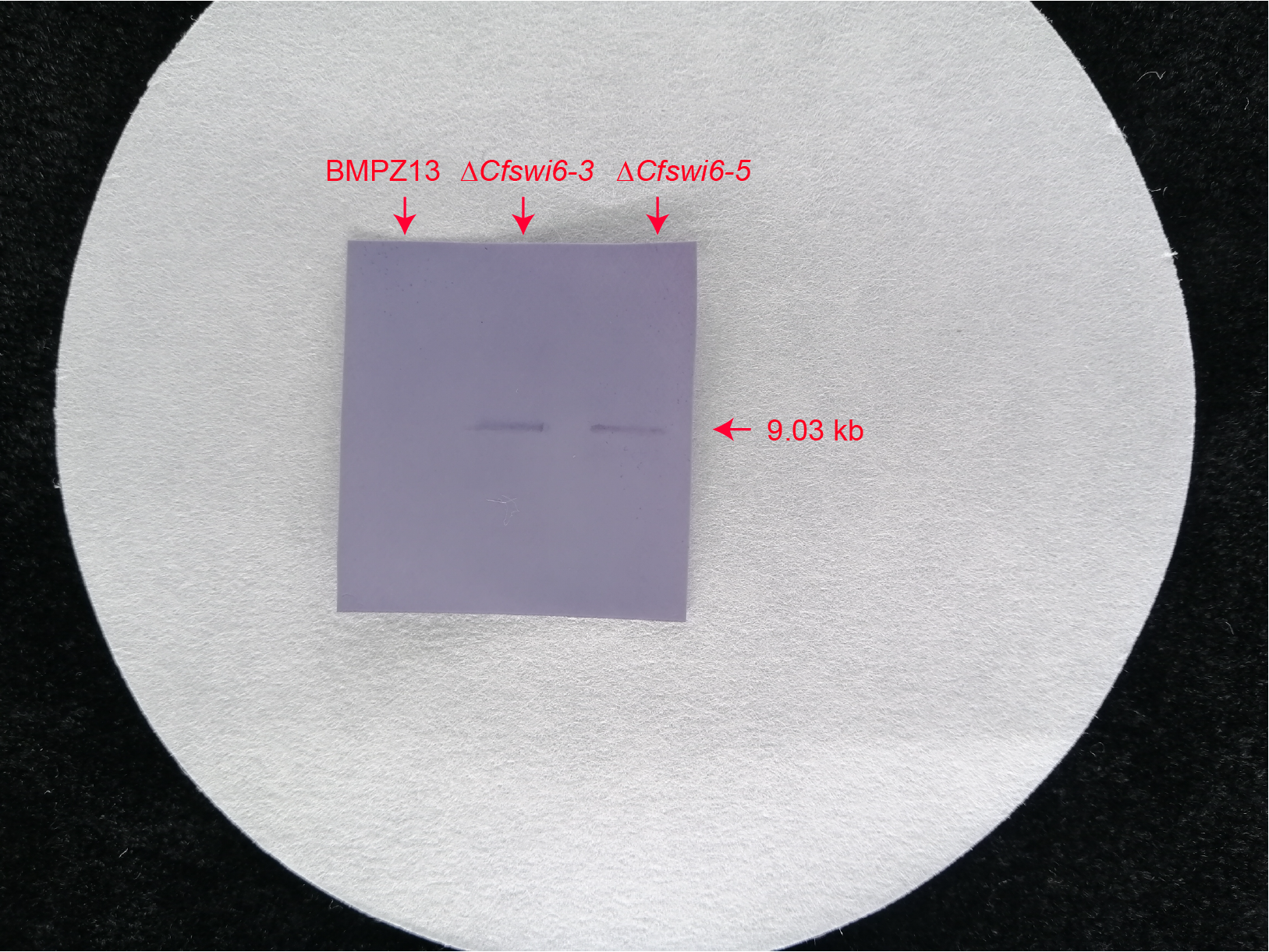

Supplement: Supplementary file 1 [file microorganisms-11-02666-s001.zip › Supplementary Figure S1/Figure S1C/Figure S1C Probe 2.tif]

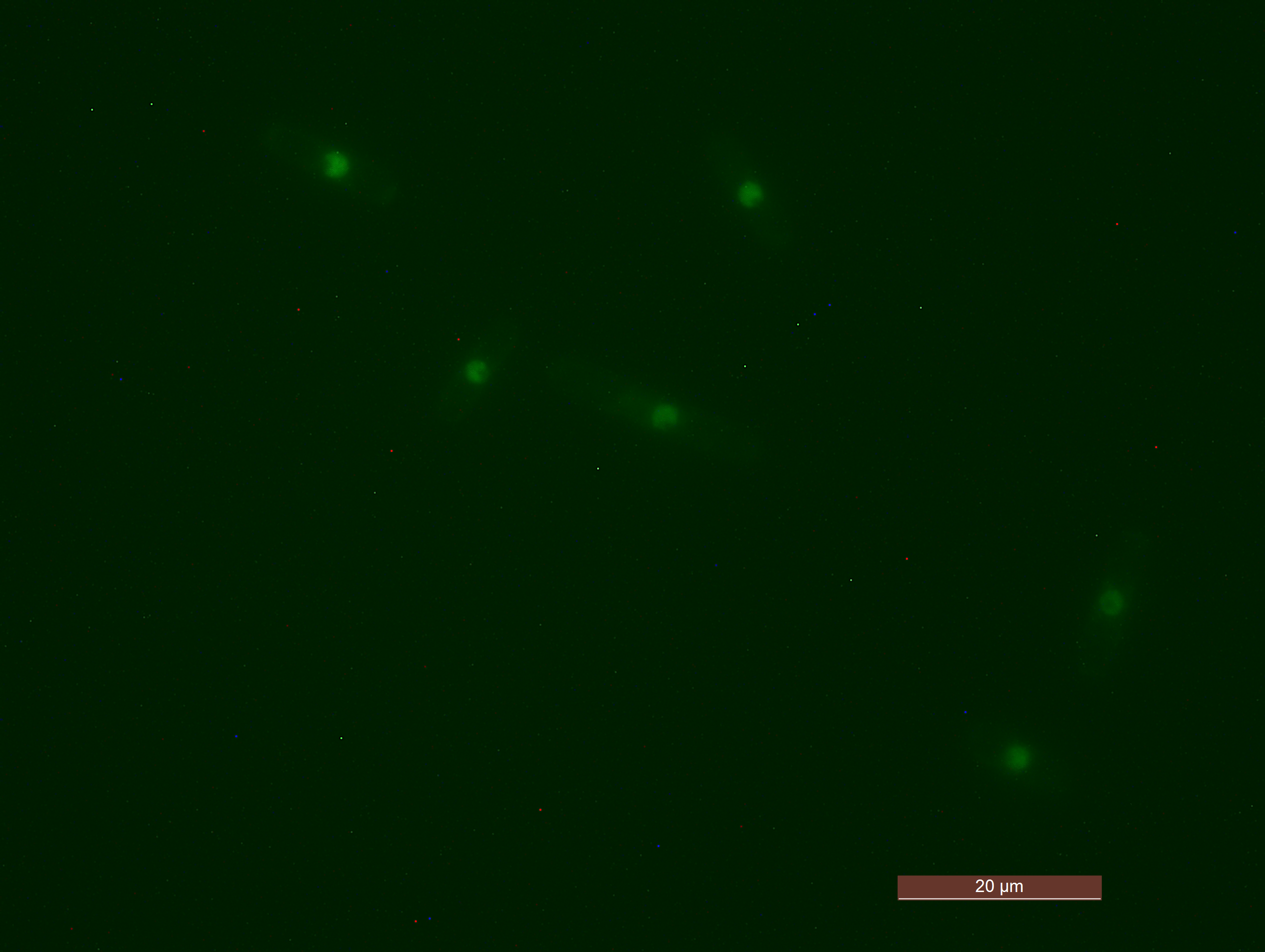

Supplement: Supplementary file 1 [file microorganisms-11-02666-s001.zip › Supplementary Figure S1/Figure S1D/Figure S1D Conidia/Figure S1D Conidia GFP.tif]

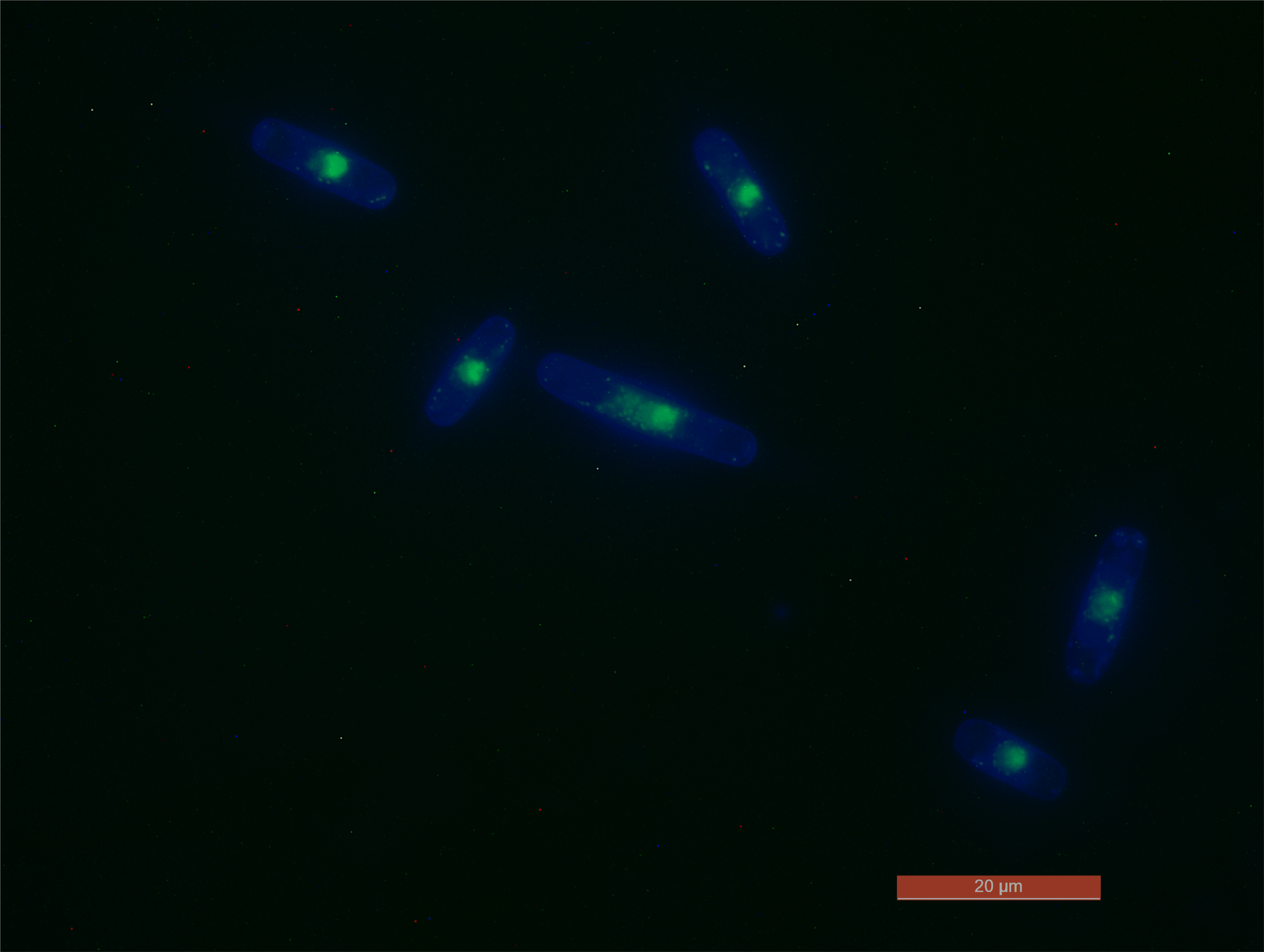

Supplement: Supplementary file 1 [file microorganisms-11-02666-s001.zip › Supplementary Figure S1/Figure S1D/Figure S1D Conidia/Figure S1D Conidi merged.tif]

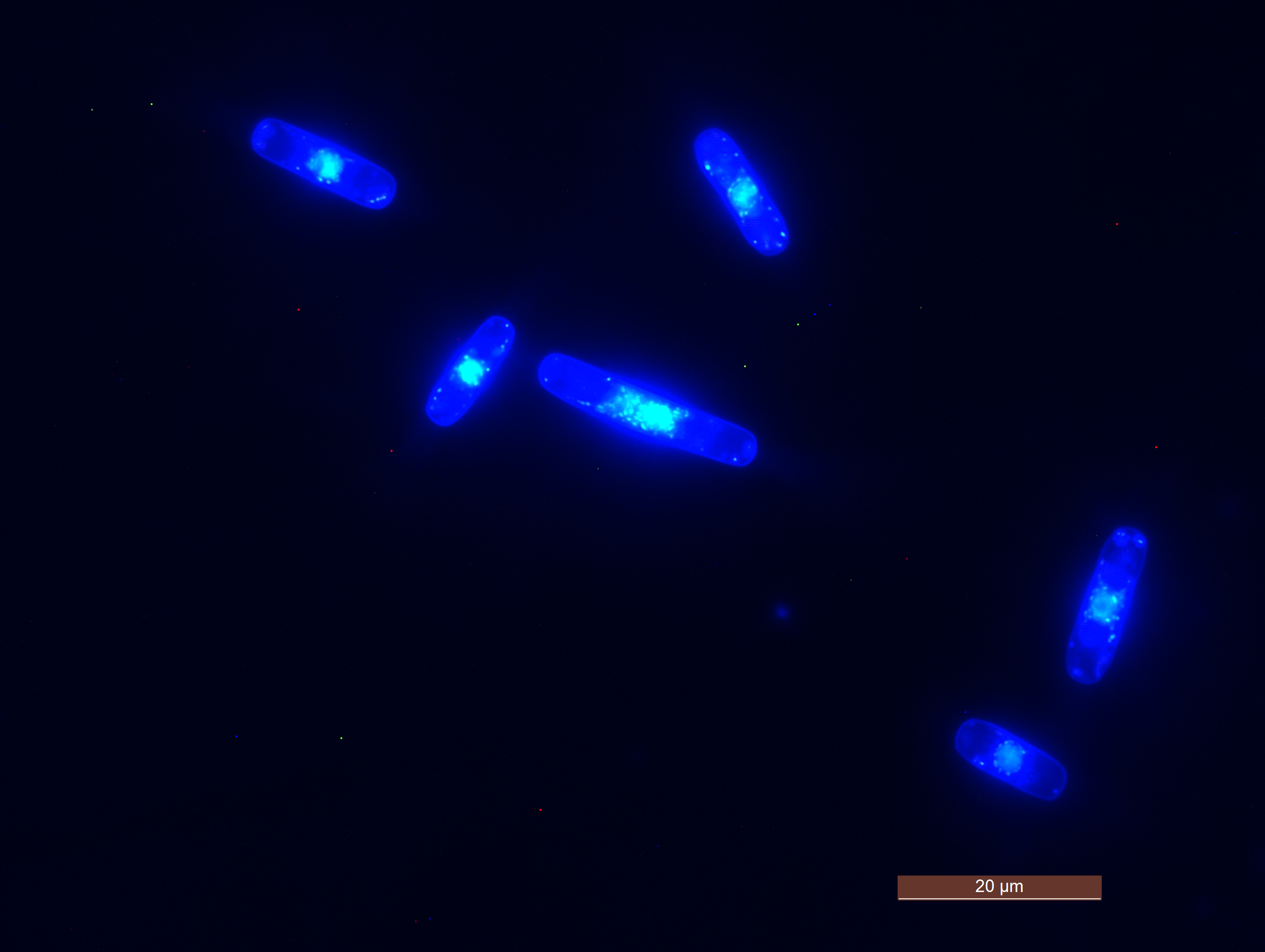

Supplement: Supplementary file 1 [file microorganisms-11-02666-s001.zip › Supplementary Figure S1/Figure S1D/Figure S1D Conidia/Figure S1D Conidia DAPI.tif]

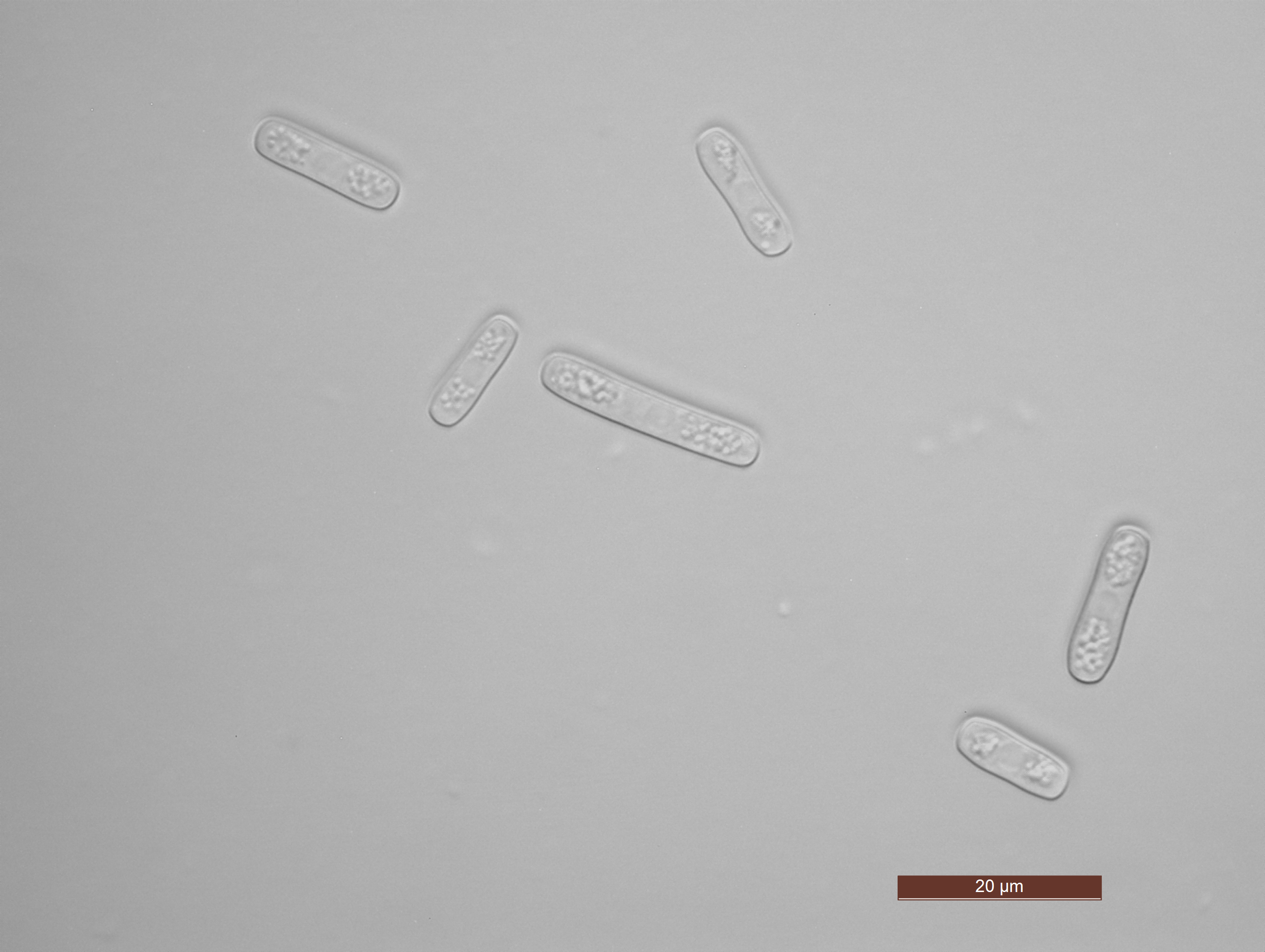

Supplement: Supplementary file 1 [file microorganisms-11-02666-s001.zip › Supplementary Figure S1/Figure S1D/Figure S1D Conidia/Figure S1D Conidia DIC.tif]

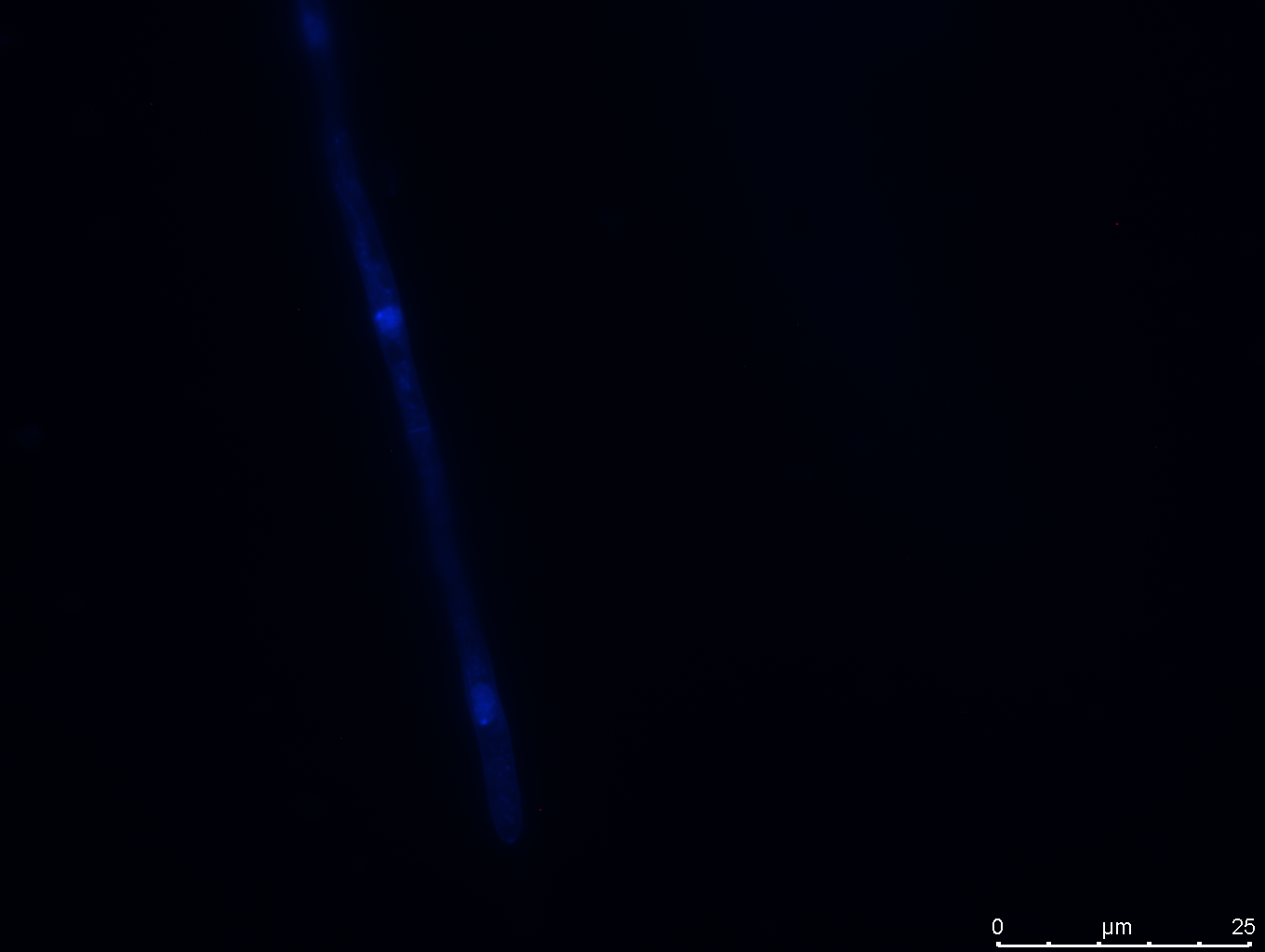

Supplement: Supplementary file 1 [file microorganisms-11-02666-s001.zip › Supplementary Figure S1/Figure S1D/Figure S1D Hypha/Figure S1D Hypha DAPI.tif]

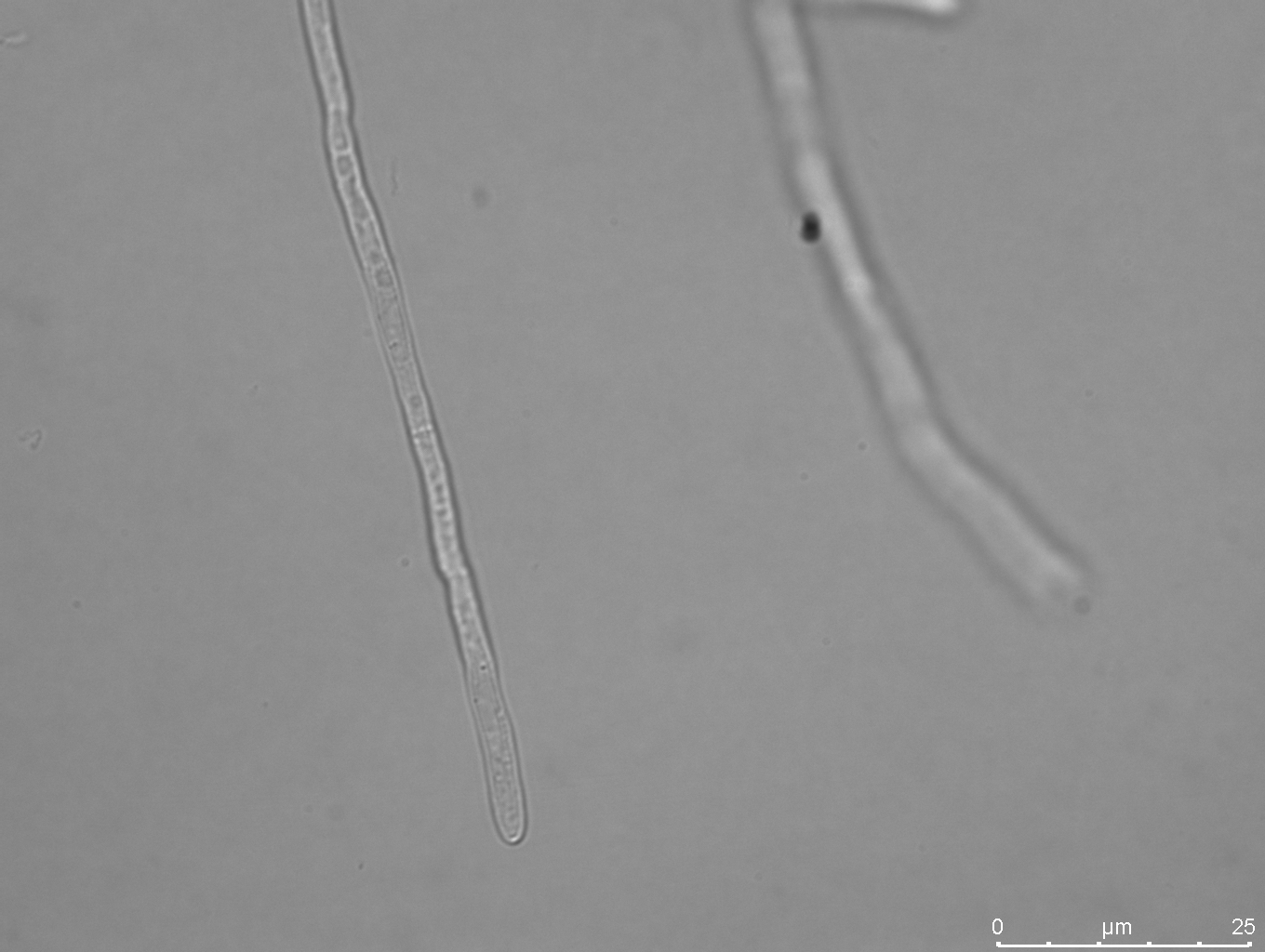

Supplement: Supplementary file 1 [file microorganisms-11-02666-s001.zip › Supplementary Figure S1/Figure S1D/Figure S1D Hypha/Figure S1D Hypha DIC.tif]

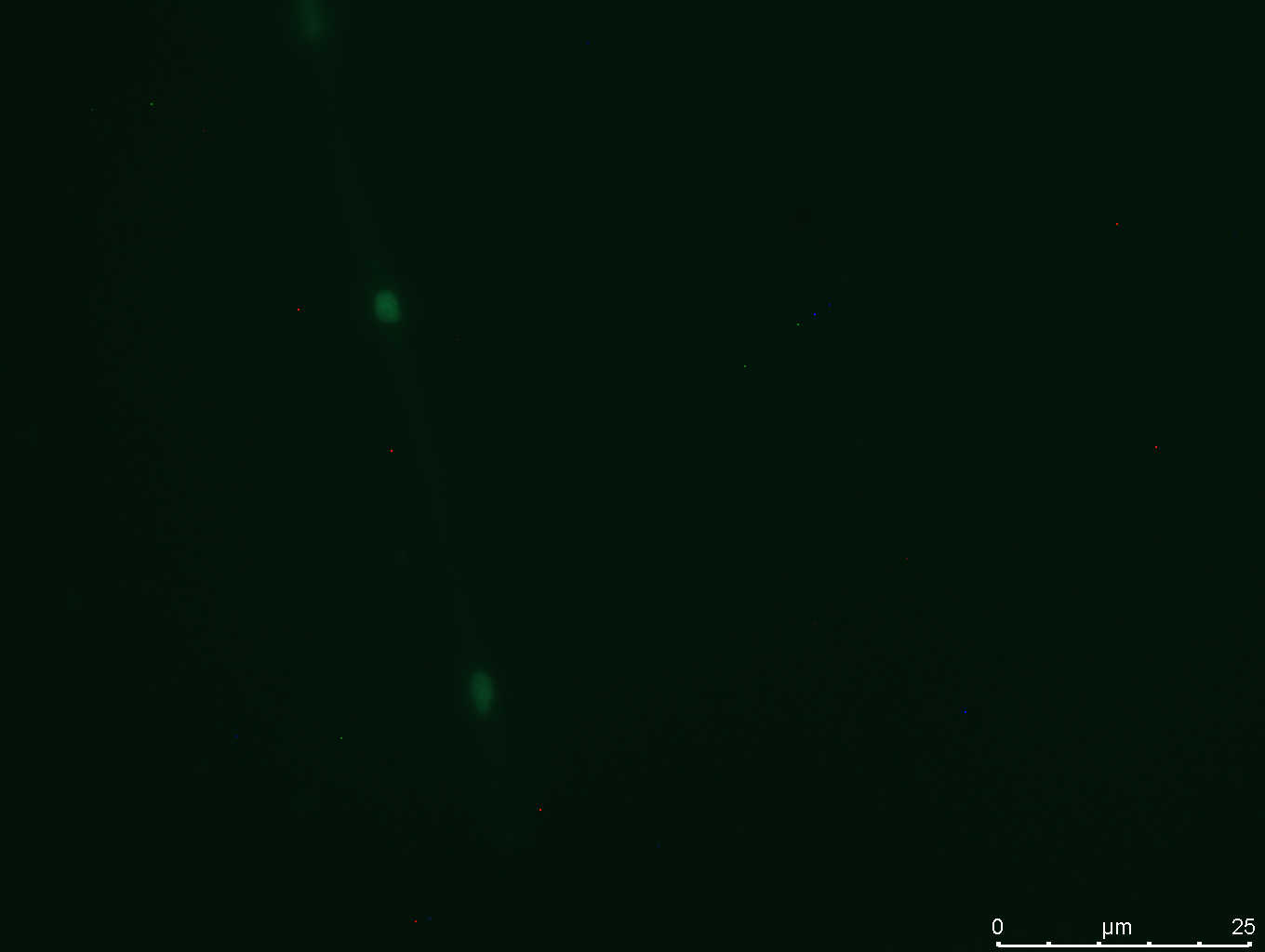

Supplement: Supplementary file 1 [file microorganisms-11-02666-s001.zip › Supplementary Figure S1/Figure S1D/Figure S1D Hypha/Figure S1D Hypha GFP.tif]

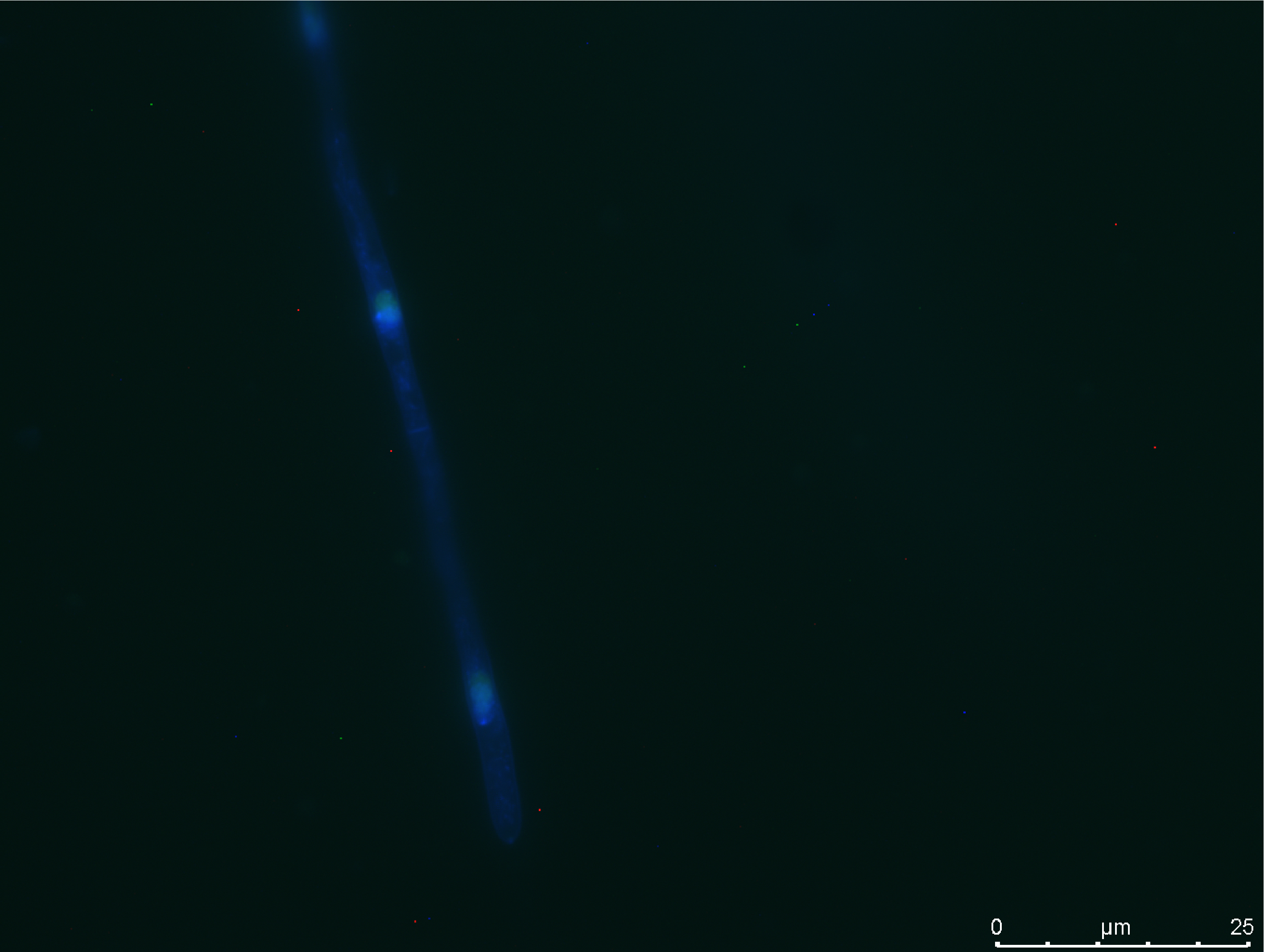

Supplement: Supplementary file 1 [file microorganisms-11-02666-s001.zip › Supplementary Figure S1/Figure S1D/Figure S1D Hypha/Figure S1D Hypha merged.tif]
